# Novel Bioresorbable Bone Wax for Potentiated Hemostasis and Osteogenesis

**DOI:** 10.1002/advs.202514616

**Published:** 2025-10-13

**Authors:** Longbao Feng, Yanbin Lu, Pengyu Fu, Shaojun Li, Pengning She, Yang xiao, Jiaming Ye, Wanqi Li, Shaochuan Li, Wei Xue, Rui Guo

**Affiliations:** ^1^ Key Laboratory of Biomaterials of Guangdong Higher Education Institutes, Key Laboratory of Regenerative Medicine of Ministry of Education, Guangdong Provincial Engineering and Technological Research Centre for Drug Carrier Development, Department of Biomedical Engineering Jinan University Guangzhou 510632 China; ^2^ College of Veterinary Medicine South China Agricultural University Guangzhou 510640 China

**Keywords:** bone hemostasis, osteogenesis, quaternized cationic starch, resorbable biomaterial, β‐TCP

## Abstract

Orthopedic surgery faces dual challenges of uncontrolled bleeding and poor bone regeneration at defect sites. While traditional beeswax‐based hemostats remain clinically prevalent, their non‐resorbability impedes healing and triggers chronic inflammation. This study introduces a “hemostasis‐resorption‐osteogenesis” synergistic design paradigm, developing a novel bioresorbable bone wax based on a polymer dispersion matrix that integrates a quaternized cationic starch (QS) and β‐tricalcium phosphate (β‐TCP) dual‐phase promoting matrix (PST). PST bone wax's physicochemical properties, characterized via finger pressing models and in vitro sealing assays, demonstrate excellent plasticity and adhesiveness, enabling rapid sealing and prolonged hemostasis under physiological blood pressure. It gradually resorbs, releasing Ca^2^⁺/PO_4_^3^^−^ ions that induce osteogenic differentiation in bone mesenchymal stem cells (BMSCs) and mineralization into hydroxyapatite (HA) crystals mimicking natural bone matrix. Hemocompatibility tests reveals its ability to adsorb erythrocytes and aggregate/activate platelets, effectively facilitating coagulation. In vivo experiments in rabbit and beagle models validate efficient hemostatic and osteogenic capabilities. Ultimately, PST bone wax achieves immediate hemostasis and long‐term osteoregeneration through QS and β‐TCP synergy, exhibiting favorable biocompatibility and safety, providing an innovative clinical solution with broad prospects.

## Graphical abstract

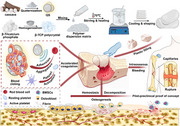

## 1 Introduction

Uncontrolled bleeding complicating bone defects poses a significant challenge in orthopedic surgery, with over 1.78 billion global fracture cases annually,^[^ ^1^, ^2^ ^]^ 30% of which require intraoperative hemostatic intervention.^[^ ^3^, ^4^ ^]^ Effective hemostasis at bone defect sites is critical for surgical success, as the vascularized nature of cancellous and cortical bone impedes spontaneous hemostasis unlike cutaneous tissues.^[^ ^5^, ^6^ ^]^ Conventional hemostatic methods such as electrocautery, compression, or packing exhibit operational limitations and risk xenogeneic protein residue or tissue trauma.^[^ ^7^, ^8^ ^]^ More critically, traditional beeswax‐based bone wax,^[^ ^9^, ^10^ ^]^ widely used in clinical settings, remains non‐degradable even unresorbable in situ, causing mechanical interference with bone regeneration and chronic inflammation due to foreign body reactions.^[^ ^11^ ^]^ With the global volume of orthopedic surgeries rising annually, the development of resorbable biomaterials combining immediate hemostasis and osteoregenerative capacity has become an urgent clinical imperative.

Current bone hemostatic materials suffer from multifaceted limitations that hinder their application in complex bone defect repair. Physically occlusive agents, such as collagen, gelatin, and fibrin,^[^ ^12^ ^]^ often fail under physiological vascular pressures due to their inherent water absorption properties and weak tissue adhesion, leading to displacement or dissolution before effective hemostasis is achieved.^[^ ^13^ ^]^ Powdered starch formulations, despite their low cost, lack sufficient moldability to conform to the irregular geometry of deep‐seated bone defects, resulting in incomplete coverage of bleeding sites and subsequent rebleeding.^[^ ^14^, ^15^ ^]^ Synthetic polymers, exemplified by polymethyl methacrylate (PMMA) bone cement,^[^ ^16^ ^]^ exhibit poor bioactivity and elicit severe inflammatory responses upon degradation, as their degradation byproducts (e.g., methyl methacrylate monomers) are cytotoxic to osteoblasts and mesenchymal stem cells.^[^ ^17^ ^]^ Traditional non‐resorbable bone wax, while effective for acute hemostasis, induces long‐term mechanical competition with regenerating bone tissue; its persistent presence disrupts the local microenvironment by triggering chronic foreign body reactions, which not only impede osteogenic cell migration and proliferation but also delay vascularization‐a critical process for bone defect repair.^[^ ^18^, ^19^ ^]^ Current partially absorbable bone waxes based on calcium phosphate cement (CPC) exhibit incomplete resorption and its cement hydration‐dependent stability frequently causing fibrous encapsulation.^[^ ^20^, ^21^ ^]^ Existing commercial absorbable bone waxes (e.g., Ostene, BoneSeal) prioritise singular hemostasis and exhibit inadequate bone healing outcomes.^[^ ^10^, ^22^ ^]^ Collectively, these limitations of existing materials underscore the need for a next‐generation hemostatic agent that can address both immediate bleeding control and long‐term osteoregeneration in a synergistic manner.

**Scheme 1:**
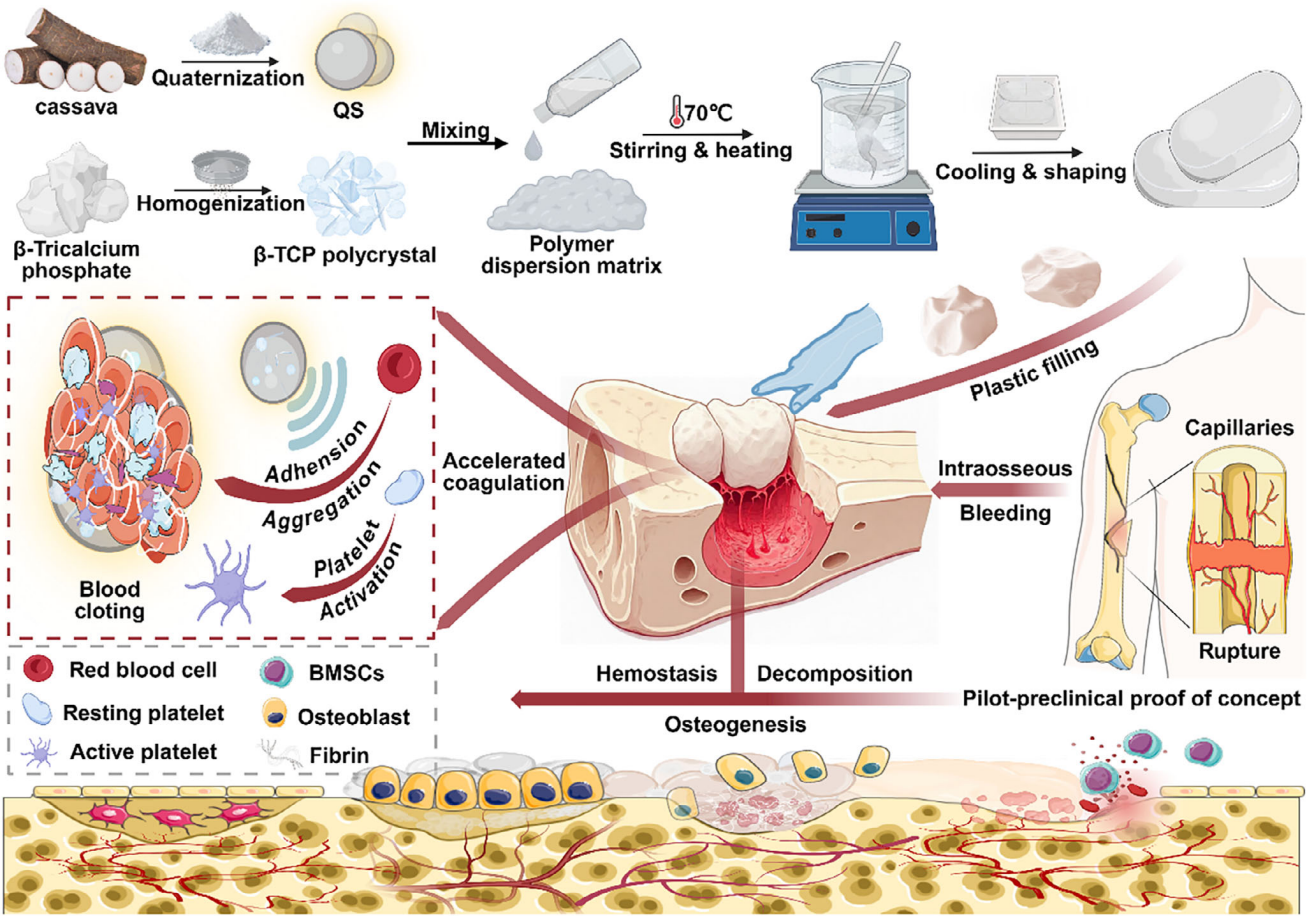
Schematic illustration of the synthesized bioresorbable bone wax material for hemostasis and osteogenesis. The preparation process of the novel resorbable bone wax and its mechanism of accelerating coagulation and promoting osteoregeneration through blood cell aggregation. Created with BioRender.com.

To address these challenges, this study introduces a “hemostasis‐resorption‐osteogenesis” synergistic design based on a QS and β‐TCP dual‐phase promoting matrix (**Scheme**  **1**). QS, derived from cassava starch via quaternization,^[^ ^23^ ^]^ features a high‐density positive surface charge,^[^ ^24^ ^]^ enabling electrostatic erythrocyte adsorption and platelet glycoprotein receptor activation to accelerate hemostasis,^[^ ^25^ ^]^ with its microporous structure concentrating coagulation factors via capillary action.^[^ ^14^, ^25^ ^]^ Complementarily, β‐TCP (Ca/P ≈ 1.5) mimics native bone mineral composition,^[^ ^26^ ^]^ releasing Ca^2^⁺/PO_4_^3^^−^ ions that stimulate BMSCs osteogenic differentiation^[^ ^27^ ^]^ Crucially, unlike CPC or α‐TCP—which form calcium‐deficient hydroxyapatite delaying early cellular/vascular infiltration^[^ ^28^ ^]^—β‐TCP degrades directly into nano‐hydroxyapatite, providing a natural scaffold for mineralization during bone remodeling.^[^ ^29^, ^30^ ^]^ Leveraging their favorable interactions with ceramic particles and biocompatible resorbable profiles,^[^ ^31^ ^]^ poloxamer 188 (P188) and polyethylene glycol‐polypropylene glycol block copolymer (PEG‐PPG) ensure uniform QS/β‐TCP dispersion, preserve moldability akin to traditional bone wax, and regulate resorbability kinetics to prevent iron overload. This composite integrates hemostatic functionality with osteogenic potential while circumventing conventional limitations, innovating in three dimensions: QS‐mediated electrostatic interactions and β‐TCP‐derived calcium ions synergize to enhance dynamic hemostasis; in situ‐formed nano‐hydroxyapatite networks replicate natural bone matrices to support cell migration/differentiation; large‐animal studies validate integrated hemostatic and osteoregenerative efficacy from acute injury to chronic repair stages. This hierarchical functional integration provides a paradigm shift for next‐generation bone hemostasis biomaterials.

## 2 Results

### 2.1 Characteristics of Synthesized PST Bone Wax

**Figure 1:**
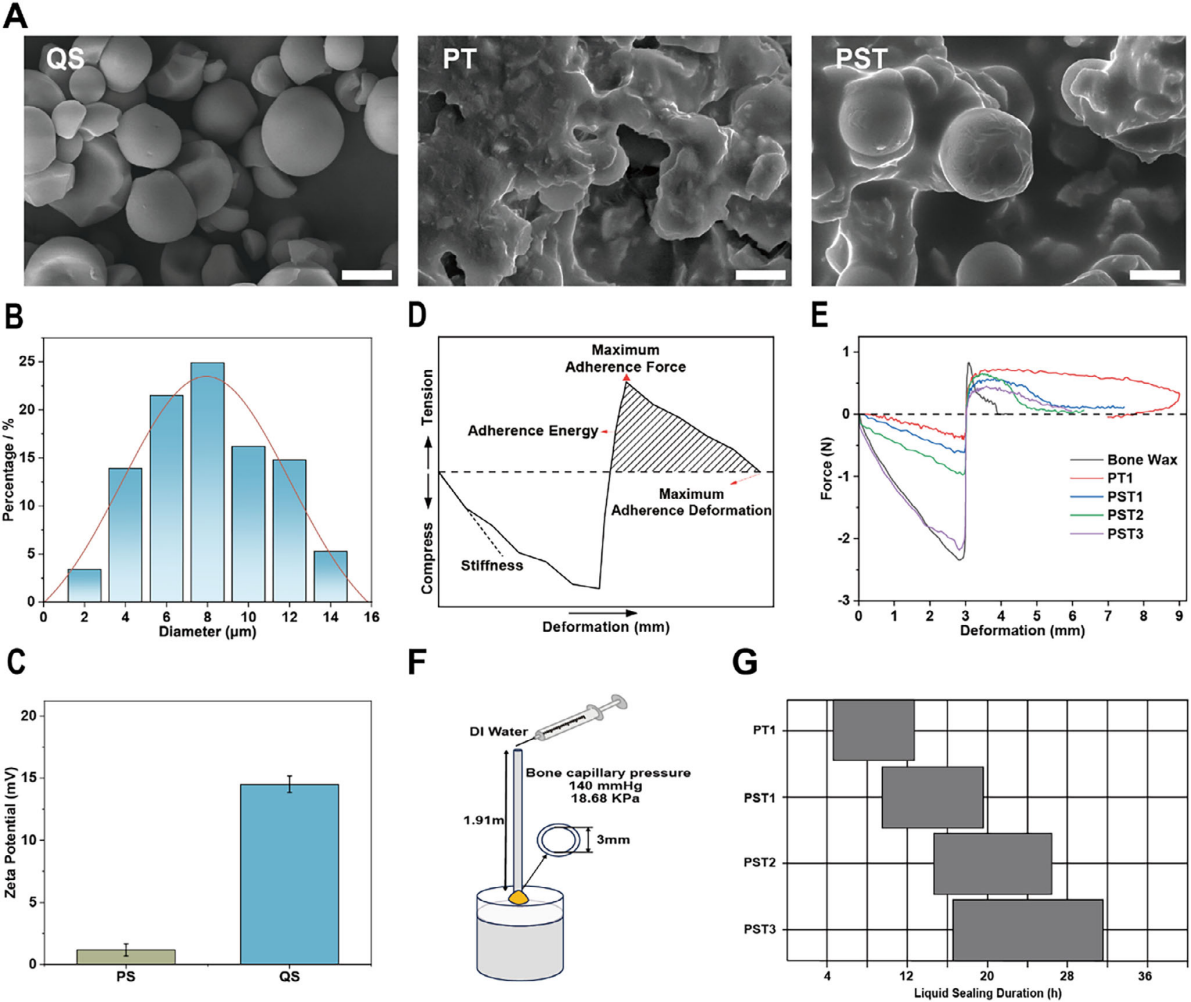
Characteristics of PST bone wax. A) Surface morphology of resorbable bone wax obtained by SEM (scale bar, 10 µm; QS: Quaternized cationic starch; PT: Bone wax with polymer dispersion matrix containing only β‐TCP; PST: Bone wax with polymer dispersion matrix containing QS and β‐TCP). B) Particle size distribution of QS. C) Zeta potential of PS and QS (PS: Pregelatinized Starch). D) Schematic diagram of texture analysis of bone wax. E) Texture analysis curve of bone wax. F) Schematic diagrams of in vitro simulation of bone capillary pressure sealing. G) Liquid sealing duration of bone wax. n ≥ 3 for each group. Error bars denote means ± SD; *p < 0.05, **p < 0.01, and ***p < 0.001.

To address the issues of continuous bleeding and poor repair capacity at bone defect sites, a novel bioresorbable bone wax with hemostatic and osteogenic capabilities was designed. The preparation of PST bone waxes involved two main steps. First, QS derived from cassava and osteoinductive β‐TCP were mixed to form a biphasic promoting matrix. Second, this biphasic matrix was thoroughly mixed with a polymer dispersion matrix and heated at 70 °C to induce pregelatinization and uniform dispersion of the starch. The mixture was then loaded into molds and cooled to shape a series of PST resorbable bone waxes for the next experiments (Scheme 1). **Figure** **1**A presents the scanning electron microscopy (SEM) images of QS, revealing its uniform spherical morphology with particle sizes ranging from 4 to 10 µm, as confirmed by particle size distribution measurements (Figure 1B). The initial β‐TCP size distribution was ≈3 µm (Figure S2A, Supporting Information), whereas the particles following homogeneous mixing with QS exhibited a slight enlargement, with a distribution ranging from 6 to 14 µm (Figure S2B, Supporting Information). The chemical structure of QS was verified using ^1^H NMR spectroscopy, which confirmed the presence of quaternary ammonium groups (Figure S1, Supporting Information). Furthermore, the zeta potential of QS was measured to be 14.5 ± 0.66 mV (Figure 1C), attributed to its quaternary ammonium groups, indicating favourable and stable positive charge properties. The original β‐TCP exhibited a slight negative charge (−2.22 mV), and the zeta potential of the particles remained at ≈+15 mV after mixing with QS (Figure S2C, Supporting Information). Consequently, these characteristics likely enhance the dispersion of QS in the polymer dispersion matrix and promote blood cells aggregation. Additionally, SEM analysis of the PST bone waxes components showed that PT bone wax exhibits a rough and non‐dense surface morphology. In contrast, the addition of QS resulted in a more homogeneous dispersion within the bone wax, leading to a rough yet dense morphology, which may facilitate the sealing of ruptured blood vessels at bone defect sites (Figure 1A).

However, when bones sustain fractures or defects, the ability to provide personalized and convenient filling at the affected site is clinically challenging and significant, corresponding to the excellent plasticity of bone wax. To this end, we performed texture analysis on PST bone waxes to simulate the resistance of the material under pressure by fingers during kneading (Figure 1D; Figure S3A, Supporting Information). As shown in Figure 1E and, the addition of QS and the incremental increase in β‐TCP content enhanced the initial stiffness and smoothness of PST bone waxes. The PST3 sample achieved an initial stiffness of 1.17 ± 0.18 N/mm, comparable to Johnson& Johnson (J&J) commercial bone wax (Figure S3C, Supporting Information). In contrast to J&J bone wax's negligible adhesion deformation, PST series bone wax showed a significant decrease in adhesion deformation from 5.32 ± 0.58 to 3.7 ± 0.4 mm with initial QS addition, and further reduced to 2.49 ± 0.46 mm with increased β‐TCP (Figure S3D, Supporting Information). While the maximum adhesion force of the PST series bone wax did not show significant differences with the initial addition of QS, it decreased to ≈0.42 N with further increased β‐TCP content (Figure S3E, Supporting Information). Adhesion energy, an indicator of overall adhesion, significantly dropped from 3.1 ± 0.6 to ≈1 mJ with the initial addition of QS and remained stable (Figure S3F, Supporting Information). Finally, all PST bone waxes could be rapidly molded into a uniform paste with sufficient spreadability for various bone fracture or defect models (Figure S3B, Supporting Information). Further analysis using Fourier Transform Infrared Spectroscopy (FT‐IR) revealed the characteristic vibrational peaks of QS (‐OH stretching around 3400 cm^−1^) and β‐TCP (‐PO_4_ base vibrations around 680 and 1100 cm^−1^) in the PST series bone wax, confirming the presence of both the biphasic promoting matrix and the polymer dispersion matrix (characterized by ‐CH stretching around 2850 cm^−1^, ‐CH bending around 1450 cm^−1^, and ‐CO stretching around 1100 cm^−1^) (Figure S4, Supporting Information). These findings suggest that the addition of QS and increased β‐TCP content improves the stiffness and reduces the adhesiveness of the PST bone waxes, aligning with the desirable plasticity and lower stickiness to surgical gloves.

Beyond providing personalized and convenient filling for bone defects, achieving hemostasis by blocking bone defects with continuous bleeding for a certain period is challenging. Our solution is to design a hemostatic and osteogenic integrated bone wax that can withstand the physiological blood pressure of ruptured bone capillaries and extend retention at the bone defect site (Scheme 1). In the bone marrow capillary embolization simulation test (Figure 1F), the liquid sealing duration of PT bone wax increased from ≈4 h to around 20 h with the initial addition of QS, and further increased to nearly 32 h with increased β‐TCP content in PST bone waxes (Figure 1G). The in vitro sealing test confirmed the favorable properties of the PST series bone wax as a bone‐filling material, highlighting the importance of blocking the physiological blood pressure in bones.

### 2.2 Resorption and Mineralization Behavior of Synthesized PST Bone Wax

**Figure 2:**
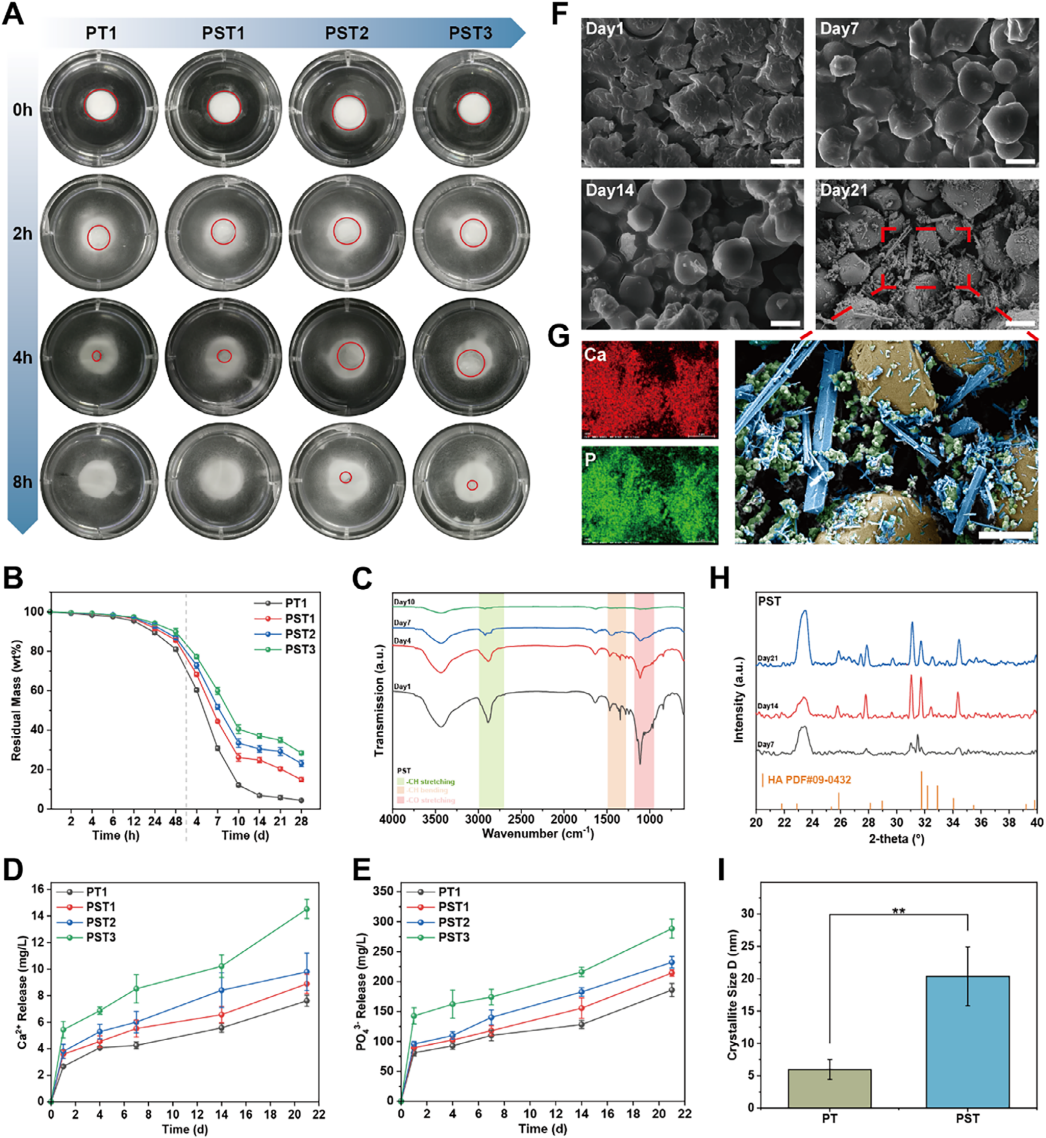
Resorption and mineralization behavior of PST bone wax. A) Solubility of bone wax in PBS (The red circle marks the bone wax that has not completely collapsed). B) Residual mass curve of bone wax during 28 days of resorption. C) FT‐IR spectra of PST bone wax during 10 days of resorption. D) Release of Ca^2^⁺ and E) PO_4_^3^^−^ during the resorption of PST bone wax. F) Residual components of PST bone wax observed by SEM after resorption for 1, 7, 14, and 21 days (scale bar, 10 µm). G) Elemental distribution and SEM pseudo‐color images of PST bone wax after 21 days of resorption (yellow: QS; green: β‐TCP; blue: hydroxyapatite mineralized from β‐TCP; scale bar, 5 µm). H) XRD of PST bone wax during 21 days of resorption, characteristic peaks of hydroxyapatite (PDF Ref. 09‐0432). I) Fitting parameters of hydroxyapatite crystalline phase based on the Korsmeyer‐Peppas model. n ≥ 3 for each group. Error bars denote means ± SD; *p < 0.05, **p < 0.01, and ***p < 0.001.

Leveraging the established properties of the PST bone waxes, we further investigated its resorption and mineralization behavior. The dissolution behavior of PST bone waxes was investigated by complete immersion in a slightly oscillating PBS buffer. The dissolution rate of the PST series bone wax slowed with the initial addition of QS and further increase in β‐TCP content, yet it fully dissolved within 8 h, indicating its complete resorbability. The addition of QS and β‐TCP effectively reduced the rapid and complete dissolution of alkylene oxide copolymers in water, preventing the disintegration of the bone wax (**Figure** **2**A). In vitro resorbable tests showed that PST bone waxes dissolved slowly by 10‐20% within 48 h and over 80% resorption by 28 days, leaving a certain amount of residual mineralized powder (Figure 2B). During the resorption process, the PBS supernatants of PST bone waxes after 1, 4, 7, and 10 days were analyzed using FT‐IR. The characteristic vibrational bonds of PEG‐PPG and P188, namely ‐CH stretching, ‐CH bending, and ‐CO stretching, were only detected up to 7 days, with a continuous decrease in transmission intensity. After 10 days, the possible PEG‐PPG and P188 residues in the PBS supernatant fell below the detection limit of the FT‐IR spectrometer, indicating that the polymer dispersion matrix of PST bone waxes resorbed almost completely within 10 days (Figure 2C; Figure S5A, Supporting Information).

Figure 2D,E shows the release of Ca^2^⁺ and PO_4_^3^^−^ ions from PST bone waxes analyzed by ICP‐MS. The release of Ca^2^⁺ showed a minimum of 2.67 ± 0.30 mg L^−1^ with no significant change with initial QS addition, but increased by 0.23≈0.43 mg L^−1^ daily with higher β‐TCP content, reaching 7.62 ± 0.42–14.53 ± 0.72 mg L^−1^ over 21 days. Similarly, PO_4_^3^^−^ release remained unchanged with initial QS addition but increased by 4.99 to 7.09 mg L^−1^ daily with increased β‐TCP content, resulting in 186.0 ± 11.07–288.60 ± 15.8 mg L^−1^ after 21 days. Additionally, the pH variation during bone waxes resorption was monitored. The pH value of PST bone waxes slightly decreased from 7.41–7.49 to 7.35–7.43 within 7 days, then returned to 7.41–7.54 over the next two weeks and remained stable. The pH changes of the PBS supernatants, where different bone wax probes were placed, were independent of the starch content. These results indicate that the pH changes occurred within a neutral range (Figure S5C, Supporting Information).

**Figure 3:**
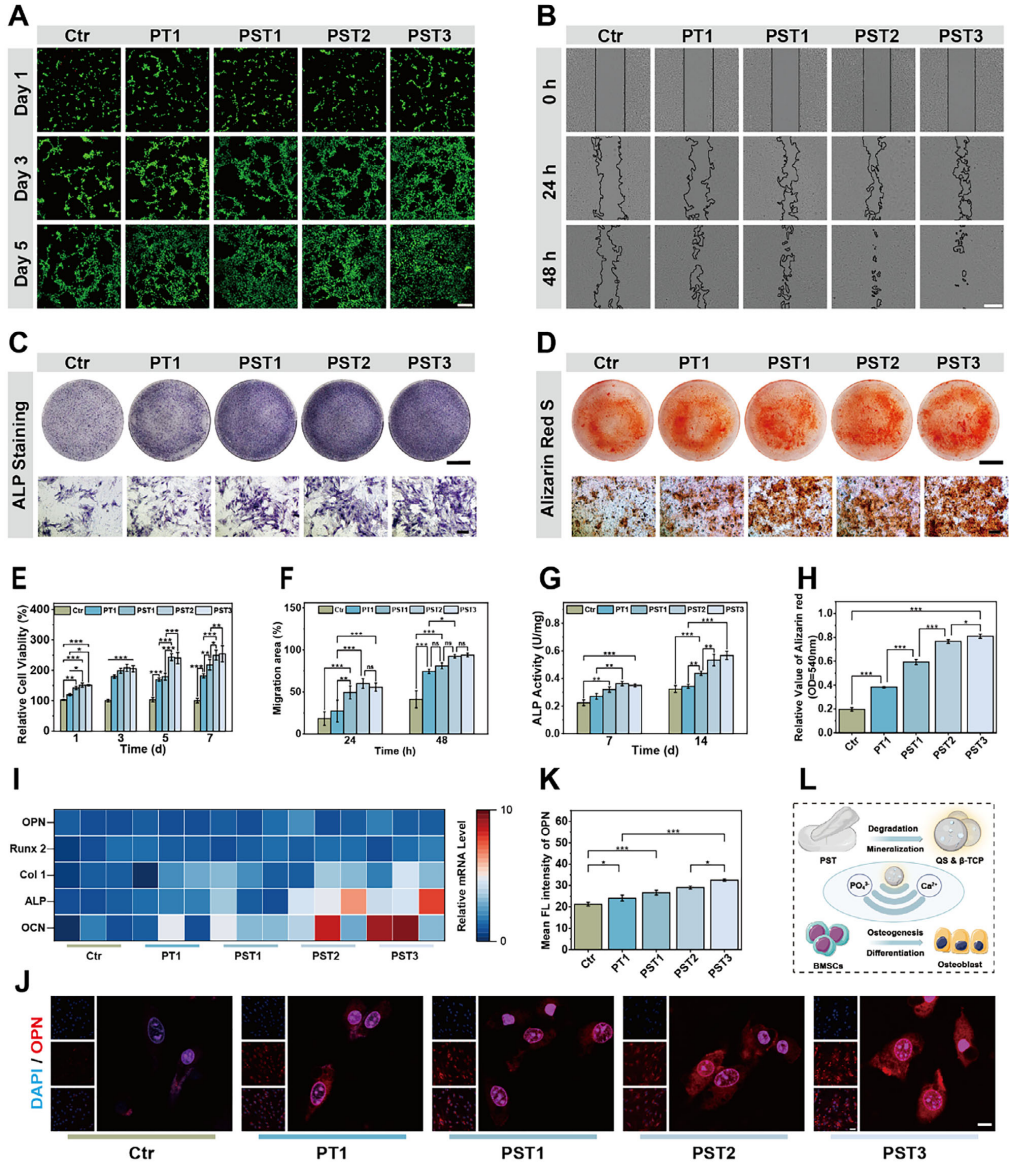
Cellular compatibility and osteogenic performance of PST bone wax. A) Live/dead assay of BMSCs cultured with bone wax extracts for 1, 3, and 5 days. Live cells appear green, and dead cells appear red (scale bar, 200 µm). B) Cell migration of BMSCs cultured with bone wax extracts for 24 and 48 h (scale bar, 400 µm). C) ALP staining of BMSCs co‐cultured with bone wax extracts for 14 days (scale bar, upper 1 cm; lower 500 µm). D) ARS staining of BMSCs co‐cultured with bone wax extracts for 21 days (scale bar, upper 1 cm; lower 500 µm). E) Cell proliferation of BMSCs cultured with bone wax extracts assessed by CCK‐8. F) Quantitative analysis of cell migration area of BMSCs cultured with bone wax extracts for 24 and 48 h. G) Quantitative analysis of ALP activity. H) Quantitative analysis of ARS‐stained calcium nodules. I) Osteogenic gene expression levels of BMSCs cultured with bone wax extracts for 14 days. J) Immunofluorescence staining of OPN in BMSCs cultured with bone wax extracts for 14 days (scale bar, 10 µm). K) Quantitative analysis of OPN fluorescence intensity. L) Schematic diagram illustrating the mechanism of bone wax in promoting BMSCs proliferation and osteogenesis. n ≥ 3 for each group. Error bars denote means ± SD; *p < 0.05, **p < 0.01, and ***p < 0.001.

Subsequently, the time‐dependent development of the phase composition of PST bone waxes were observed using SEM, further confirming the resorption process where a trace amounts of 1 to 3 µm particles are released from the solution and the polymer dispersion matrix gradually disappeared, leaving residues of QS and β‐TCP (Figure 2F; Figure S5D, Supporting Information). Notably, SEM analysis of PST bone waxes residues at 21 days showed homogenized QS (yellow) with pores, along with aggregated nanocrystals (green), more crystal phases (blue) such as rod‐like, lacerated plate‐like, and needle‐like crystals, and coverage on QS. EDS mapping of Ca and P confirmed calcium phosphate as the main component (Figure 2G). XRD analysis further revealed that at 7, 14, and 21 days, characteristic reflections of the product (HA) were visible, with product peak intensities increasing over time, indicating the ongoing mineralization of PST bone waxes into HA with diverse crystal phases during degradation (Figure 2H). Another Surprisingly, Scherrer equation calculations revealed that HA crystal sizes in PST bone waxes increased significantly from 5.96 ± 1.5 to 20.37 ± 4.56 nm with initial QS addition (Figure 2I). XRD patterns also showed significantly enhanced HA reflection intensities after 21 days of degradation, suggesting QS accelerated PST bone waxes mineralization into HA beneficial for bone repair (Figure S5B, Supporting Information). Overall, these results demonstrate that the resorption and mineralization of PST bone waxes play crucial roles in initial hemostasis at bone defect sites and subsequent bone regeneration processes (**Figure** 3).

### 2.3 Cytocompatibility and Osteogenic Performance of PST Bone Wax

Building on the established physicochemical properties and resorbability behavior of PST bone waxes, we further evaluated their biological performance by examining cytocompatibility and osteogenic properties. BMSCs were indirectly co‐cultured with different PST bone waxes to assess cytocompatibility. Initially, the cytocompatibility of PST bone waxes were determined using live/dead cell staining on BMSCs after 1, 3, and 5 days of treatment. All bone wax groups exhibited excellent cytocompatibility with minimal dead cells, and cell proliferation density increased with higher β‐TCP (Figure 3A). Subsequently, cell viability of BMSCs treated with PST bone waxes for up to 7 days were assessed using the CCK‐8 assay. Compared to the blank control, the relative cell viability of BMSCs treated with PST bone waxes were significantly enhanced, increasing with higher β‐TCP (Figure 3E). Similarly, the migration area of BMSCs within 48 h of PST bone waxes treatment showed that increased β‐TCP content promoted cell migration, further confirming the good cytocompatibility and cell viability enhancement of PST bone waxes components (Figure 3B,F).

**Figure 4:**
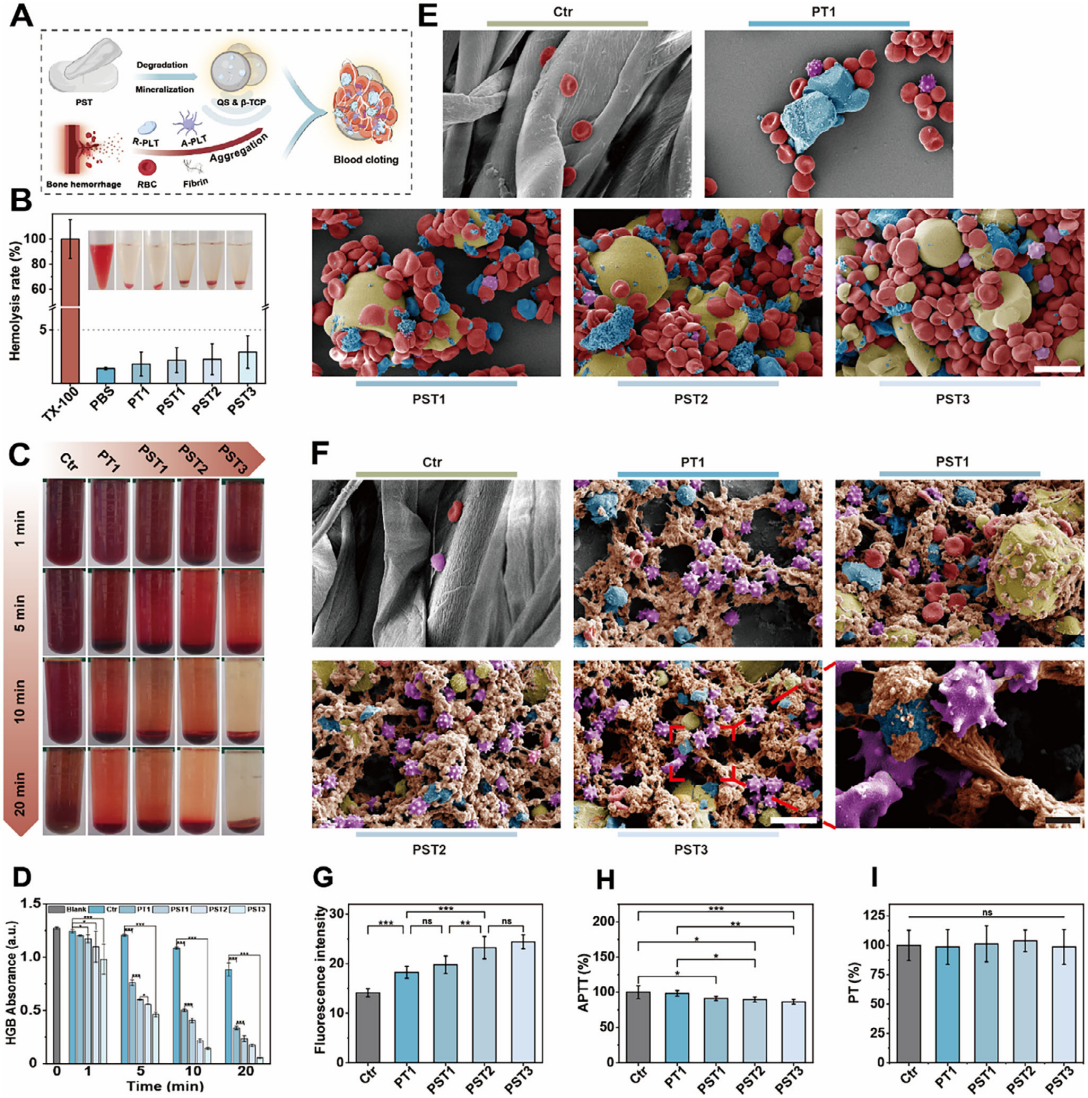
Blood compatibility and blood cell aggregation properties of PST bone wax. A) Schematic illustration of the PST bone wax in promoting RBCs aggregation and PLTs activation. B) Hemolysis test of PST bone wax (TX‐100: Triton X‐100). C) Aggregation of RBCs after 20 min co‐incubation with PST bone wax in RBCs suspension. D) HGB content in the supernatant after 20 min co‐incubation of PST bone wax with diluted RBCs suspension. E) SEM pseudo‐color images of RBCs aggregation by PST bone wax (scale bar, 10 µm). F) SEM pseudo‐color images of PLT aggregation and activation by PST bone wax (scale bar, 10 µm) and with its localized zoomed‐in image (scale bar, 2 µm; red: RBC, blue: β‐TCP, yellow: QS, purple: PLT, orange: fibrin‐coated β‐TCP). G) Intracellular Ca^2^⁺ fluorescence intensity of activated PLTs by PST bone wax. H) APTT test of PST bone wax. I) PT test of PST bone wax. n ≥ 3 for each group. Error bars denote means ± SD; *p < 0.05, **p < 0.01, and ***p < 0.001.

To assess the efficiency of calcium mineral deposition and osteogenic differentiation of PST bone waxes, alkaline phosphatase (ALP) staining and Alizarin Red S (ARS) staining were performed, representing early osteoblast differentiation and inorganic calcium, respectively. Results showed that PST bone waxes with added QS and increased β‐TCP content significantly induced ALP activity in BMSCs, particularly in the PST3 group (Figure 3C,G). Similarly, PST bone waxes facilitated the formation of more mineralized nodules, with the highest mineralization degree observed in the PST3 group (Figure 3D,H). To further confirm the osteogenic‐promoting effects of PST bone waxes, RT‐PCR was used to analyze the expression of osteogenic differentiation‐related genes in BMSCs. Results demonstrated that PST bone waxes upregulated the expression of Runt‐related transcription factor 2 (RUNX2), Osteopontin (OPN), Osteocalcin (OCN), Collagen type I (COL1), and ALP in a β‐TCP concentration‐dependent manner, with initial QS addition further enhancing this upregulation (Figure 3I). Immunofluorescence staining for the osteogenic marker protein OPN in BMSCs yielded consistent results (Figure 3J,K). Collectively, these findings suggest that PST bone waxes enhance cell viability and stimulates osteogenesis in BMSCs through Ca^2+^ and PO_4_^3−^ release (Figure 2D,E and Figure 3L).

### 2.4 Blood Compatibility and Coagulation Properties of PST Bone Wax

Based on the established cellular compatibility and osteogenic performance of PST bone waxes, we further evaluated their blood compatibility and ability to aggregate blood cells. **Figure** **4**A illustrates the hemostatic course of PST bone waxes, which aggregates red blood cells (RBCs) and activates platelets (PLTs) via the positive charge of QS and Ca^2+^ released from β‐TCP, leading to clot formation. Additionally, blood proteins were involved in the PLTs adhesion/activation and RBCs aggregation assays to simulate the blood clotting process. Direct co‐culturing of RBCs or PLTs with PST bone waxes were conducted. The blood compatibility of PST bone waxes was determined by hemolysis assays, which revealed low hemolysis ratios (<5%) for all PST bone waxes (Figure 4B), confirming their good blood compatibility. The in vitro impact of different PST bone waxes on whole blood clotting were evaluated by monitoring the number of RBCs not engaged in clots, as measured by the absorbance of hemoglobin (HGB) in the supernatant. All bone waxes showed procoagulant effects, with clearer supernatants than the control group throughout the clotting process. As the addition of QS and β‐TCP increased, the samples demonstrated greater blood aggregation within the same time frame, indicated by clearer supernatants and lower absorbance values (PST3 < PST2 < PST1 < PT1). Notably, PST3 achieved complete clotting within 20 min, as evidenced by absorbance values approaching zero and nearly transparent supernatants, highlighting its robust procoagulant activity. In contrast, the procoagulant effect of PT bone wax without QS was significantly weaker than that of PST bone waxes, underscoring the crucial role of QS in enhancing procoagulant activity (Figure 4C,D).

Further, SEM was used to observe the effects of PST bone waxes on RBCs aggregation and PLTs adhesion/activation. Compared to the control group of gauze with only sporadic RBCs adhesion, the PT group showed more RBC aggregation near β‐TCP, indicating its ability to aggregate RBC. Remarkably, PST bone waxes with added QS exhibited significantly greater RBC aggregation than the control and PT groups, with the extent of aggregation increasing with higher β‐TCP (Figure 4E). Similarly, the PT group demonstrated substantial PLTs adhesion and activation around β‐TCP compared to the gauze. The addition of QS in PST bone waxes significantly increased PLTs adhesion compared to the PT group. The PLTs progressed from a resting “biconvex” discoid shape to an activated “stellate” dendritic form with pseudopodia, then to a spread morphology with pseudopodia extending and spreading over the QS surface. Finally, they formed a “honeycomb” structure with interwoven pseudopodia and a fibrin network around QS&β‐TCP, indicating PST bone waxes' ability to activate and aggregate PLTs (Figure 4F). PST bone waxes also showed significantly lower non‐adherent platelet numbers (measured by lactate dehydrogenase content), further confirming their superior PLTs congregation ability (Figure S6, Supporting Information). Additionally, after incubation with PLTs, PST groups exhibited higher fluorescence intensity (indicating intracellular Ca^2^⁺ levels) than the control and PT groups, showing greater platelet activation (Figure 4G). Collectively, these results highlight PST bone waxes' superior performance in PLTs adhesion/activation and RBCs aggregation.

The in vitro hemostatic property of PST bone waxes was further assessed by blood‐clotting index (BCI) assay. PST bone waxes and medical gauze (control group) were incubated with citrated whole blood (without CaCl_2_) or recalcified whole blood (with CaCl_2_) to measure BCI values, reflecting the materials' dependence on or independence from the coagulation system.^[^ ^14^ ^]^ As shown in Figure S7 (Supporting Information), medical gauze exhibited high BCI values without CaCl_2_ and low BCI values with CaCl_2_, indicating moderate hemostatic properties in regular bleeding but low efficacy in severe bleeding, consistent with its clinical use for minor hemorrhage.^[^ ^32^ ^]^ In contrast, all PST bone waxes showed lower BCI values than the control gauze, regardless of CaCl_2_ presence, and the values decreased further with increased QS and β‐TCP content. Interestingly, the BCI values of PST2 and PST3 were similar regardless of CaCl_2_, indicating their hemostatic properties were independent of the coagulation system. Most importantly, the BCI values of PST3 were significantly lower than those of other groups, highlighting its superior hemostatic performance among the PST bone waxes. Finally, activated partial thromboplastin time (APTT) and prothrombin time (PT) tests were performed on PST bone waxes. Compared to the control group and PT bone wax, the addition of QS and increased β‐TCP led to a significant shortening of APTT, while PT showed no significant difference. This suggests that the QS and β‐TCP components of PST bone waxes may synergistically enhance activation of the intrinsic coagulation pathway (Figure 4H,I).

### 2.5 In Vivo Hemostasis and Osteogenesis Promotion by PST Bone Wax

**Figure 5:**
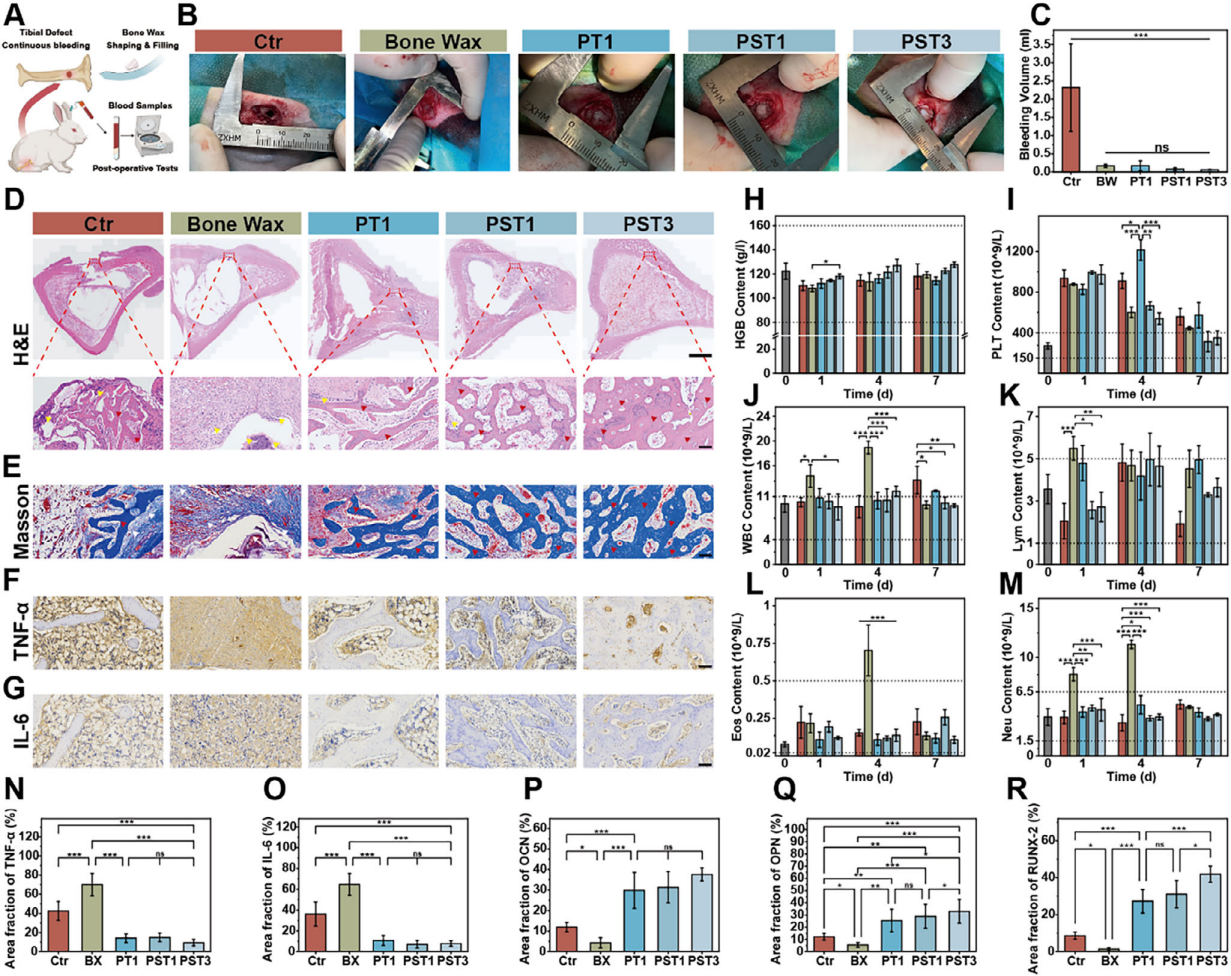
Treatment of PST bone wax in a rabbit cancellous bone defect model. A) Schematic illustration of PST bone wax application in a rabbit tibial bone defect model. B) Intraoperative digital images of hemostasis at the bone defect site using different bone waxes. C) Blood loss within 3 min of bone wax application at the defect site. D) H&E staining of bone sections after 4 weeks of treatment with different bone waxes (scale bar, 1000 µm) and magnified views of the defect area (scale bar, 100 µm; yellow triangles, inflammatory cells; red triangles, new bone tissue). E) Masson trichrome staining of bone sections after 4 weeks (scale bar, 100 µm; white triangles, myofibers). F) and G) Immunohistochemical staining of bone sections after 4 weeks of treatment. H) Content changes in HGB, I) PLT, J) white WBC, K) Lym, L) Eos, and M) Neu in whole blood samples within 7 days post‐treatment. N) TNF‐α, O) IL‐6, P) OCN, Q) OPN, and R) RUNX2 immunohistochemical staining quantification. n ≥ 3 for each group. Error bars denote means ± SD; *p < 0.05, **p < 0.01, and ***p < 0.001.

In vitro results showed PST bone waxes has good hemostatic properties and promotes BMSCs osteogenic differentiation. Subsequent in vivo studies assessed its hemostasis and osteoregeneration capabilities. First, a rabbit tibial cancellous bone defect model was established, and blood samples were collected immediately post‐surgery and within the first week (**Figure** **5**A). During the procedure, bone waxes application instantaneously halted bleeding at the defect site. Compared to the gauze control group, which exhibited substantial blood loss of 2.32 ± 1.2 mL within 3 min, all bone wax groups showed negligible blood loss. Notably, while commercial J&J bone wax exhibited marginal bleeding at the defect site 3 min post‐application, all PST bone waxes groups successfully achieved hemostasis within 3 min without failure. This superior performance is attributed to the excellent plasticity and adhesiveness of PST bone waxes, which effectively seals bone marrow fissures and sinusoids, blocking blood flow and terminating bone tissue bleeding (Figure 5B,C). Subsequently, to investigate the continuous bleeding behavior before long‐term bone repair, rabbit whole blood samples were collected on postoperative days 1, 4, and 7. Low HGB levels are typically associated with postoperative bleeding or anemia.^[^ ^14^, ^33^ ^]^ All groups maintained HGB levels within the normal range of 80–160 g L^−1^. Notably, the PST3 group exhibited significantly higher HGB levels than the blank and other bone wax groups on days 4 and 7 (Figure 5H), indicating better HGB recovery/production, likely due to reduced blood loss during healing. Higher PLT levels initially enhance clotting at wound sites.^[^ ^9^ ^]^ All groups showed elevated PLT levels on postoperative day 1, aiding primary hemostasis. Over time, PLT levels decreased toward the normal range of 150–400 × 10⁹ L^−1^. The PST1 and PST3 groups showed the fastest recovery, suggesting they better maintain normal blood status after addressing persistent bleeding. Interestingly, the PT1 group exhibited the highest PLT levels on postoperative day 4, possibly due to transient secondary bleeding at the wound site (Figure 5I). Additionally, changes in white blood cell (WBC) lineage levels were monitored to assess postoperative inflammation.^[^ ^9^, ^34^, ^35^ ^]^ The gauze control group showed a significant increase in WBC. Moreover, the commercial J&J bone wax group exhibited elevated levels of WBC, lymphocytes (Lym), eosinophils (Eos), and neutrophils (Neu) beyond the normal physiological range. In contrast, PT, PST1 and PST3 bone waxes maintained WBC lineage levels within the normal range (Figure 5J–M), indicating good biocompatibility and no short‐term immune rejection.

To further evaluate the bone repair promotion of PST bone waxes during long‐term osteoregeneration, tibial samples from rabbits were collected at 4 and 8 weeks post‐surgery for histological and radiological analysis. As shown in Figure 5D,E, initial histological assessment via H&E and Masson staining indicated a significant presence of inflammatory cells (marked with yellow triangles) at the bone defect site in both the blank control and commercial J&J bone wax groups at week 4, with partial inflammation still evident at week 8. However, the PT, PST1, and PST3 bone waxes all showed minimal inflammatory cells. Notably, the non‐resorbable J&J bone wax led to pronounced fibrous tissue encapsulation (indicated by white triangles), persisting through week 8 and hindering new bone formation. In contrast, all the PST bone waxes groups demonstrated a clear promotion of bone regeneration at the lesion site. At week 4, newly formed trabecular bone in the PT1, PST1, and PST3 groups exhibited a porous network structure with irregular surface pores (marked with red triangles), with the PST3 group showing the densest bone network. By week 8, this structure matured into highly dense cortical bone (Figure S8, Supporting Information). Quantitative immunohistochemical analysis of osteogenic markers OCN, OPN, and RUNX2^[^ ^36^ ^]^ revealed significantly higher expression levels in the PST bone waxes groups compared to the blank and commercial bone wax groups (Figure S9, Supporting Information; Figure 5P–R), further confirming the superior bone regeneration capability of PST bone waxes.

Subsequently, the expression of inflammation and immune‐regulation related proteins/cytokines, tumor necrosis factor‐α (TNF‐α) and interleukin‐6 (IL‐6), was evaluated.^[^ ^14^ ^]^ The non‐resorbable J&J bone wax group exhibited marked TNF‐α and IL‐6 expression at week 4. The gauze control also showed some TNF‐α and IL‐6 expressions, likely due to persistent bleeding at the bone defect site before long‐term bone repair. In contrast, the PT, PST1 and PST3 bone waxes demonstrated no significant expression (Figure 5F,G). Quantitative analysis revealed that the J&J bone wax group had significantly higher percentages of TNF‐α and IL‐6 positive areas than the gauze and PST3 bone wax groups (Figure 5N,O). These immunohistochemical results were consistent with the H&E staining analysis (Figure 5D), indicating that the PST groups elicited minimal inflammatory or immune responses during osteoregeneration. Finally, the PST bone waxes showed normal ranges in hematological and biochemical parameters (Figure S10, Supporting Information). H&E staining of the heart, liver, spleen, lung, and kidney also revealed normal structures (Figure S11, Supporting Information). These findings strongly support the biocompatibility and biosafety of PST bone waxes. Their excellent bioactivity may be attributed to their efficient resorption under physiological conditions and the effective in situ mineralization and osteogenesis promoted by their biphasic components.

**Figure 6:**
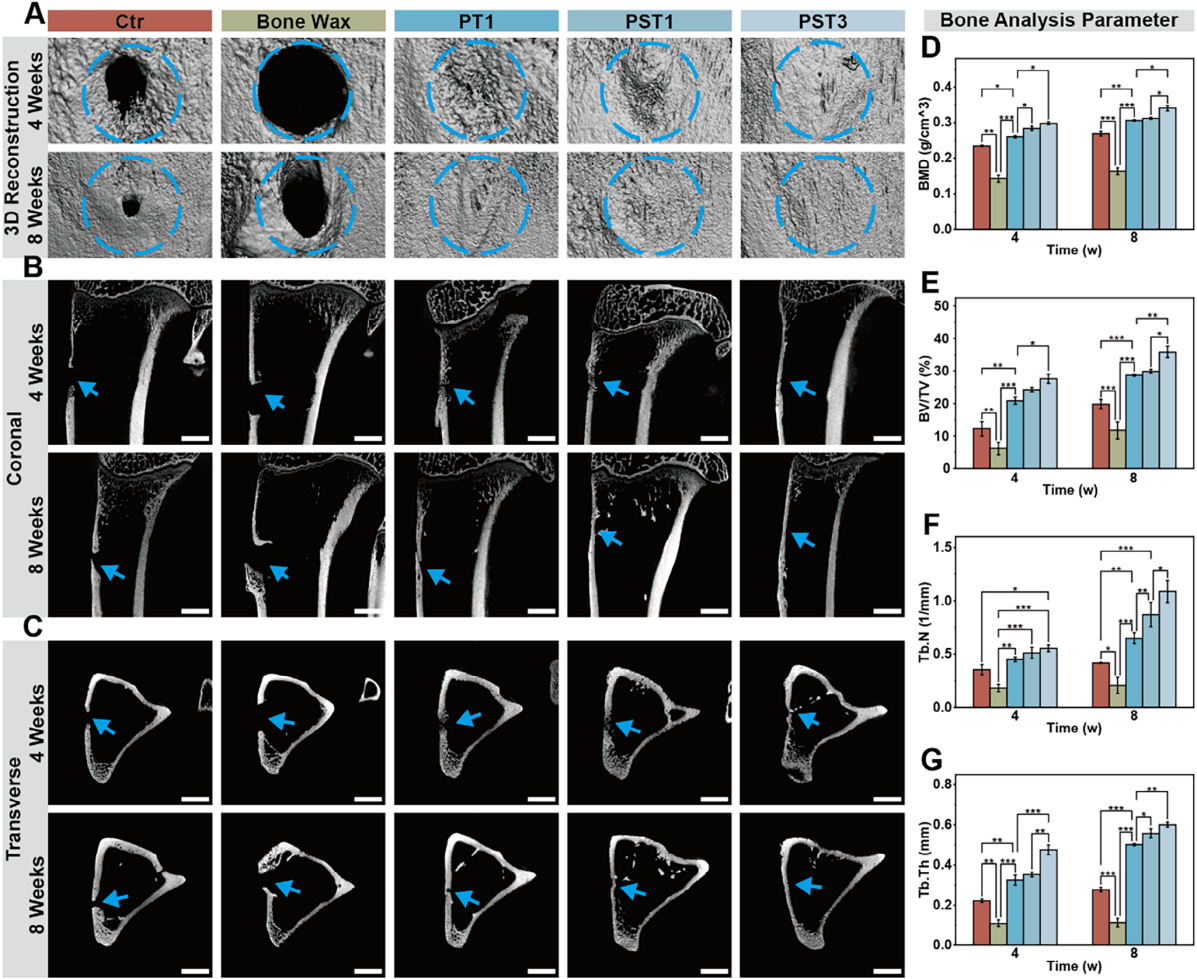
Radiological evaluation of the bone regeneration ability of PST bone wax. A) 3D reconstructed micro‐CT images of bone defect sites in rabbit tibias at 4 and 8 weeks post‐implantation (blue circle, 4 mm in diameter). B) Coronal and C) transverse micro‐CT images of the tibial bone defect samples (scale bar, 4 mm; blue arrow, bone defect area). D) Bone mineral density (BMD), E) bone volume/total volume (BV/TV), F) trabecular number (Tb.N), and G) trabecular thickness (Tb.Th) analyses based on micro‐CT. n ≥ 3 for each group. Error bars denote means ± SD; *p < 0.05, **p < 0.01, and ***p < 0.001.

Micro‐computed tomography (micro‐CT) analysis of rabbit tibiae demonstrated that from postoperative week 4, all groups exhibited progressive new bone ingrowth from defect margins toward the center, except the J&J bone wax group which showed persistent bone voids and even ectopic bone proliferation at week 8 (**Figure** **6**A). Notably, PST3 bone wax achieved complete defect healing with dense bone formation by week 8, significantly outperforming the control, PT1, and PST1 groups. Furthermore, coronal/transverse sections revealed markedly reduced defect areas and increased bone density in PST groups compared to the control group's unresolved cavities (Figure 6B,C), with gross specimens confirming smooth, intact surfaces in PST‐treated defects (Figure S12, Supporting Information). Quantitatively, PST bone waxes showed content‐dependent osteogenic enhancement with increasing QS and β‐TCP content: PST3 yielded significantly higher bone mineral density (BMD) at 4 weeks (0.30 ± 0.01 g cm^−^^3^) and 8 weeks (0.34 ± 0.01 g cm−^3^) versus Blank (0.23 ± 0.02 and 0.27 ± 0.01 g cm−^3^; Figure 6D), while bone volume fraction (BV/TV), trabecular number (Tb.N) and trabecular thickness (Tb.Th) were consistently elevated (Figure 6E–G). Collectively, these data validated PST bone waxes' superior bone osteogenic capacity.

**Figure 7:**
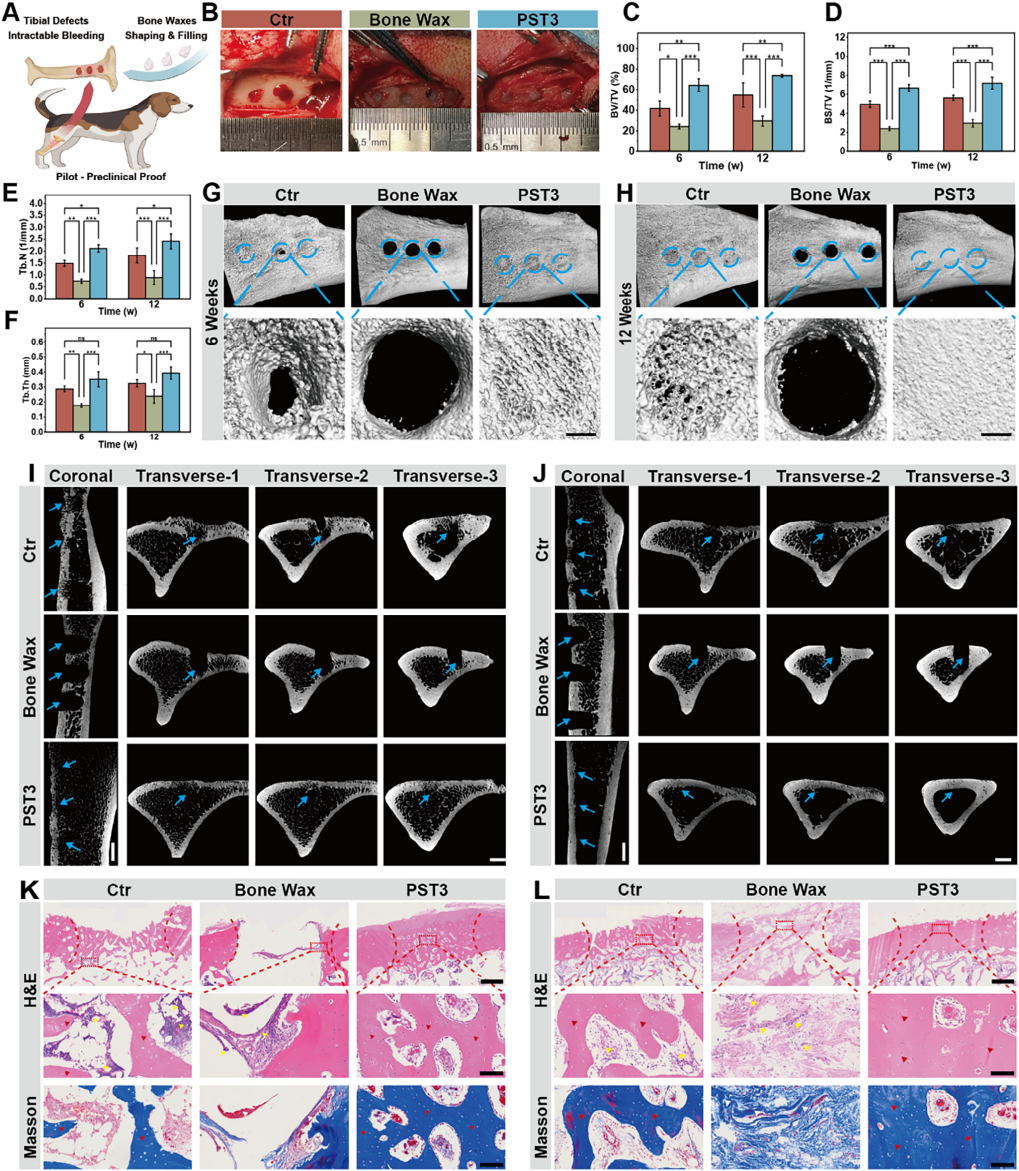
Treatment of PST bone wax in a beagle cancellous bone defect model. A) Schematic illustration of PST bone wax application in a beagle tibial bone defect model. B) Intraoperative digital images of hemostasis at the bone defect site using different bone waxes. C) BV/TV, D) bone surface to total volume ratio (BS/TV), E) Tb.N, and F) Tb.Th analyses based on micro‐CT. G) 3D reconstructed micro‐CT images of bone defect sites in beagle tibias at 6 and H) 12 weeks post‐implantation (blue circle, 4 mm in diameter) with magnified views of the defect area (scale bar, 1 mm). I) and J) Coronal and transverse micro‐CT images of the tibial bone defect samples at 6 and 12 weeks (scale bar, 4 mm; blue arrow, bone defect area). (K) H&E and Masson trichrome staining of bone sections after 6 weeks and (L) 12 weeks of treatment with different bone waxes (scale bar, 1000 µm) and magnified views of the defect area (scale bar, 100 µm; yellow triangles, inflammatory cells; red triangles, new bone tissue; white triangles, myofibers). n ≥ 3 for each group. Error bars denote means ± SD; *p < 0.05, **p < 0.01, and ***p < 0.001.

To validate the hemostatic and osteoregenerative efficacy of PST bone waxes in a clinically relevant large mammal model, beagle cancellous bone defects were established as a pivotal pilot‐preclinical investigation (**Figure** **7**A). Notably, during intraoperative hemostasis assessment, the PST3 group exhibited minimal blood loss (0.69 ± 0.47 mL within 3 min), significantly outperforming gauze controls (5.0 ± 0.5 mL) and J&J bone wax (1.6 ± 0.62 mL) (Figure S13, Supporting Information). Critically, under elevated physiological blood pressure inherent to large‐mammal defects, PST3 achieved complete hemorrhage control within 3 min without material disintegration, whereas J&J bone wax showed marginal bleeding and gauze controls demonstrated persistent hemorrhage at the defect site (Figure 7B). Subsequently, wound exudate volume was quantified to evaluate persistent hemorrhage progression. At postoperative day 1, PST3 yielded significantly lower exudate volumes (2.1 ± 0.30 mL) versus J&J bone wax (3.8 ± 0.81 mL) and blank control (8.6 ± 0.56 mL). Furthermore, this reduction persisted through day 7 (PST3: 0.3 ± 0.27 mL vs blank: 5.1 ± 0.71 mL; Figure S14, Supporting Information), confirming PST3's efficacy in mitigating intractable bone‐defect hemorrhage. Consistently, pronounced swelling attributable to chronic hemorrhage and tissue fluid accumulation was observed in blank controls at day 7, with mild swelling in the J&J group and absence thereof in PST3‐treated defects (Figure S15, Supporting Information).

To evaluate osteoregeneration, beagle tibiae with defects were harvested at weeks 6 and 12 for micro‐CT analysis. Representative 3D reconstructions demonstrated progressive defect healing across groups (blank, J&J bone wax, and PST3) from weeks 6 to 12 (Figure 7G,H). Notably, PST3‐treated defects exhibited robust trabecular bridging that formed a dense interconnected network, culminating in complete cortical coverage by week 12. Conversely, blank controls displayed exposed defects at week 6, with only loosely arranged trabeculae partially filling defects by week 12. Critically, J&J bone wax hindered osteoregeneration, showing no new bone formation due to its non‐resorbability. Further micro‐CT analysis of transverse planes revealed a persistent bone void in blank controls at week 6 (Transverse‐2 section), with limited periosteal callus formation only in Transverse‐1 and ‐3 sections. In contrast, PST3 achieved superior defect‐filling density in both coronal and transverse sections (Figure 7I,J). Consistently, quantitative bone morphometry confirmed significantly higher new bone volume and mineral density in PST3 versus controls (Figure 7C–F). Gross examination of harvested tibiae corroborated these findings: blank controls retained visible defects, while J&J group showed ectopic fibrous tissue encapsulation. Strikingly, PST3 generated smooth cortical surfaces without excessive ectopic tissue proliferation (Figure S16, Supporting Information), attributable to its bioresorbability and intrinsic osteogenic capacity.

Following debridement of peri‐tibial musculature, harvested bone samples underwent histological analysis with H&E and Masson staining. As illustrated in Figure 7K,L, J&J bone wax elicited substantial inflammatory‐associated cell infiltration (yellow triangles) at both 6 and 12 weeks, demonstrating poor biocompatibility and progressive fibrous encapsulation (white triangles). Conversely, PST3‐treated defects exhibited minimal inflammation at week 6 due to inherent bioresorbability, advancing to normal tissue morphology (ordered cellular architecture devoid of inflammatory cells) and forming intricately woven bone structures (red triangles) by week 12. Collectively, these histopathological findings corroborate PST3's multifunctional efficacy in large‐mammal cancellous defects‐integrating rapid hemostasis, osteoregeneration, and biosafety—thereby demonstrating significant clinical translation potential.

## 3 Discussion

This study successfully developed a novel bioresorbable bone wax that simultaneously addresses intraoperative hemostasis and long‐term osteoregeneration in bone defects through a biphasic matrix design incorporating QS and β‐TCP. Notably, in a large‐mammal model, PST3 demonstrated exceptional intraoperative hemostatic efficacy (Figure S13, Supporting Information) and achieved complete defect bridging with cortical coverage by 12 weeks (Figure 7G,H). Critically, PST3 maintained structural integrity under high physiological blood pressure – 120 to 160 mmHg^[^ ^37^, ^38^, ^39^ ^]^ (Figure 7B), while its resorbability eliminated the fibrous encapsulation and chronic inflammation associated with commercial bone waxes, offering a important solution for clinical bone hemostasis management.

Materially, the incorporation of naturally derived QS and β‐TCP optimized the PST series’ texture properties: Texture analysis revealed enhanced initial stiffness (1.17 ± 0.18 N mm^−1^ for PST3, Figure 1E), comparable to J&J bone wax (Figure S3C, Supporting Information), alongside reduced adhesive energy (from 3.1 ± 0.6 to ≈1 mJ, Figure S3F, Supporting Information). This enabled rapid intraoperative molding and tight sealing of irregular defects (Figure 5B; Figure S3B, Supporting Information), while minimizing surgical glove adherence^[^ ^40^, ^41^ ^]^ (adhesive deformation decreased from 5.32 ± 0.58 to 2.49 ± 0.46 mm, Figure  S3D, Supporting Information). Mechanistically, the dense surface morphology observed via SEM (Figure 1A) provided a physical barrier for vascular sealing, maintaining structural stability under high‐pressure blood flow (Figure 7B), thereby overcoming the leakage limitations of fibrin glues reported in prior studies.^[^ ^42^, ^43^ ^]^

**Figure 8:**
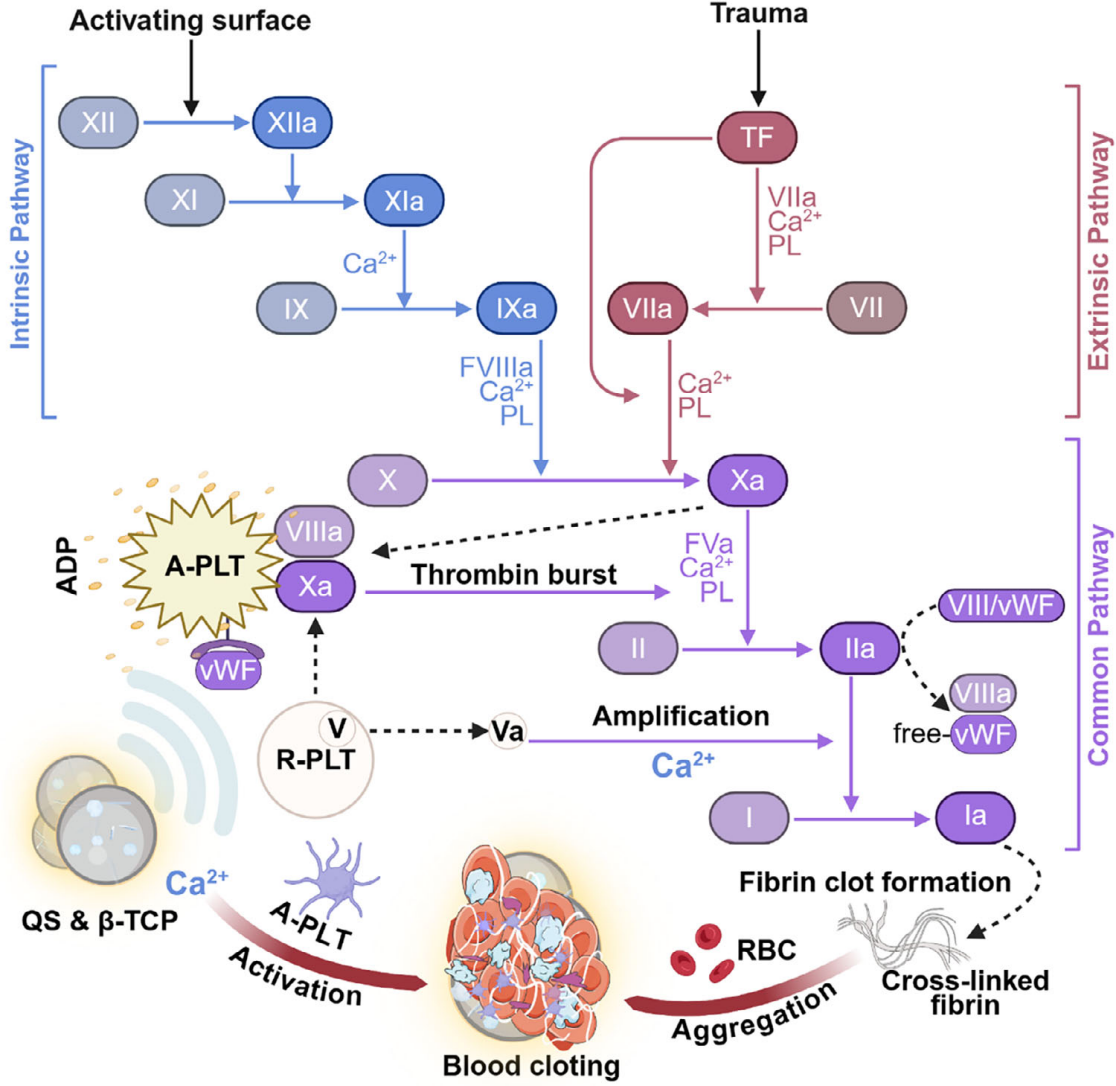
PST bone wax leverages its charged surface and controlled calcium release to rapidly initiate, propagate, and amplify the entire coagulation cascade locally at the bone bleeding site, resulting in superior, leak‐proof hemostasis. Created with BioRender.com.

PST bone waxes likely achieves superior hemostasis through a multistage coagulation cascade (**Figure** **8**): During coagulation initiation, the positively charged QS surface (Figure 1C) adsorbs tissue factor (TF) and bone collagen, facilitating TF‐VIIa complex formation and X→Xa conversion;^[^ ^44^ ^]^ concurrently, QS activates platelets, driving their transformation from biconcave discs to dendritic/honeycomb morphologies (Figure 4F, purple/orange) with enhanced phosphatidylserine exposure and ADP release.^[^ ^45^ ^]^ In propagation, Ca^2^⁺ released from β‐TCP (Figure 2D) serves as a key cofactor, activating the platelet‐surface VIIIa‐Xa complex and catalyzing a “thrombin burst.” During amplification, Ca^2^⁺ mediates fibrinogen crosslinking into fibrin, forming dense clots on the PST bone waxes surface.^[^ ^15^ ^]^ This multilevel synergy translated into: Immediate and long‐term hemostasis in large mammals (Figure 7B); Shortened APTT (Figure 4H); Near‐zero exudate (0.3 ± 0.27 mL) by postoperative day 7, eliminating edge leakage (Figure S14, Supporting Information).

Furthermore, PST facilitates bone regeneration through a well‐orchestrated “resorption‐mineralization‐cell activation” cascade. The polymer matrix is completely resorbed within 10 days (Figure 2C), leaving residual QS/β‐TCP granules that nucleate apatite' (Figure 2G). Notably, QS demonstrated enhanced ions adsorption capacity, potentially attributed to its electrostatic interactions,^[^ ^46^, ^47^ ^]^ sequestering more Ca^2^⁺/PO_4_^3^^−^ released by β‐TCP (cumulative 288.60 ± 15.8 mg L^−1^ over 21 days, Figure 2E). The microporous architecture of these residual components likely functioned as nucleation sites, directing ion organization and providing an optimal substrate for crystal nucleation and growth.^[^ ^48^ ^]^ This integrated process resulted in the in‐situ formation of abundant, larger nanohydroxyapatite crystals (20.37 ± 4.56 nm, Figure 2I), effectively establishing a biomimetic bone matrix. Consequently, this mineralized microenvironment directly promoted osteogenic differentiation, evidenced by 2.5‐fold upregulation of RUNX2/OPN expression (Figure 3I) and 40% increase in ALP activity (Figure 3G), ultimately leading to the formation of a dense trabecular network in vivo (Figure 7G). In stark contrast, the non‐resorbable J&J bone wax induced fibrous encapsulation and inflammatory responses (Figure 5D), which completely suppressed osteogenic activity (Figure 7K).

The biosafety of PST stems from its bioresorbable components and systematic validation. QS, a glucose polysaccharide derivatives, inherits low immunogenicity and metabolizable from natural polysaccharides. β‐TCP, a clinically established bone substitute, maintains physiological pH (7.35–7.54, Figure  S5F, Supporting Information), preventing acidic degradation‐induced inflammation. Experimental verification demonstrated: Hemocompatibility with hemolysis ratio <5% and absence of erythrocyte membrane damage (Figure 4B); systemic safety evidenced by postoperative hematological parameters within normal ranges (Figure 5H–M; Figure S10, Supporting Information); and preserved parenchyma of major organs without pathological lesions (Figure S11, Supporting Information).

PST redefines bone‐hemostatic biomaterials by translating a “hemostasis‐resorption‐osteogenesis” cascade from concept to clinic‐verified reality: QS surface instantaneously nucleates tissue factor and platelets through an electrostatic–chemical dual interaction that we newly delineate, while β‐TCP synchronously supplies Ca^2^⁺/PO_4_^3^^−^ to sustain a “thrombin burst”, thereby converting bleeding bone margins into a stable, apatite‐seeding clot that is gradually resorbed and replaced by host bone. This single‐material paradigm eliminates the historical trade‐off between rapid seal and bio‐functionality, surpassing passive beeswax‐type fillers, non‐resorbable CPC and hemostat‐only agents. Moldable like conventional bone waxes, PST conforms intimately to cancellous trabeculae and seals the defect without slippage, blocking low‐pressure marrow extravasation and relieving surgeons of concerns over delayed oozing of blood or tissue fluid. Every constituent is pharmacopeia‐grade and already approved for human use, removing regulatory bottlenecks and enabling immediate, cost‐effective scale‐up—an industrial advantage absent in most next‐generation biomaterials still trapped in small‐animal proof‐of‐concept stages. Validation in a stringent beagle tibial‐defect model (Figure 7) confirms that PST is a ready‐to‐use, resorbable bone waxes that simultaneously stops bleeding and orchestrates osteoregeneration, offering surgeons an economical and safer solution for orthopedic hemostasis.

## 4 Conclusion

In summary, PST bone waxes employ a hierarchical mechanism initiated by QS‐induced cationic accumulation of blood components, progressing via Ca^2^⁺‐mediated thrombin burst, and culminating in in situ mineralization‐osteogenesis, thereby synchronizing immediate hemostasis with long‐term bone regeneration in large mammals. Its optimized rheology and controlled resorption offer a key solution integrating surgical handling, reliable hemostasis, and bioactivity for clinical bone defect repair. Future efforts will address current limitations by integrating antibiotics or osteogenic factors (e.g., bone morphogenetic protein 2) for compromised bone healing and developing injectable PST bone waxes with delivery devices for minimally invasive surgery.

## Conflict of Interest

The authors declare no conflict of interest.

## Supporting information



Supporting Information

## Data Availability

The data that support the findings of this study are available from the corresponding author upon reasonable request.
