# Supplementary material for: Novel Bioresorbable Bone Wax for Potentiated Hemostasis and Osteogenesis
Source: Adv Sci (Weinh). 2025 Oct 13;13(2):e14616. doi: 10.1002/advs.202514616 (PMC12786301; doi:10.1002/advs.202514616)
Supplement: Supplementary file 1 — Supporting Information [file ADVS-13-e14616-s001.docx]

**Supporting Information**

**Novel Bioresorbable Bone Wax for Potentiated Hemostasis and Osteogenesis**

Longbao Feng ^1, #^, Yanbin Lu ^1, #^, Pengyu Fu ^1^, Shaojun Li ^1^, Pengning She ^2^, Yang xiao ^2^, Jiaming Ye ^2^, Wanqi Li ^2^, Shaochuan Li ^2, *^, Wei Xue ^1, *^, Rui Guo ^1, *^

^1^ Key Laboratory of Biomaterials of Guangdong Higher Education Institutes, Key Laboratory of Regenerative Medicine of Ministry of Education, Guangdong Provincial Engineering and Technological Research Centre for Drug Carrier Development, Department of Biomedical Engineering, Jinan University, Guangzhou, 510632, China.

^2^ College of Veterinary Medicine, South China Agricultural University, Guangzhou 510640, China.

^#^ These authors contributed to the work equally and should be regarded as co-first authors.

^*^ Co-corresponding author.

E-mails: guorui@jnu.edu.cn (Rui Guo); shaochuan@scau.edu.cn (Shaochuan Li); weixue_jnu@aliyun.com (Wei Xue)

**This PDF file includes:**

**Materials and experiments;**

**Table S1 and S2;**

**Fig. S1 to S16.**

**1. Materials and experiments**

**Materials and reagents:** The quaternized cationic starch (QS) was supplied by Guangdong Wengjiang Chemical Reagent Co., Ltd., while pregelatinized cassava starch was purchased from Shanghai Yuanye Bio-Technology Co., Ltd. β-Tricalcium phosphate (β-TCP) was obtained from Suzhou Ding'an Technology Co., Ltd. Poloxamer 188 (P188) and poly (ethylene glycol-ran-propylene glycol) (PEG-PPG) were sourced from Sigma-Aldrich. Cetyltrimethylammonium chloride (CTAC, 98%) and anhydrous ethanol (AR) were provided by Shanghai Maclin Biochemical Technology Co., Ltd. Fetal bovine serum (FBS) and Dulbecco's modified eagle medium (DMEM) were purchased from Thermo Fisher Scientific (USA), whereas sterile phosphate buffered saline (PBS), penicillin-streptomycin, and trypsin were obtained from Gibco. Osteogenic induction reagents, including β-glycerophosphate, ascorbic acid, and dexamethasone, were sourced from Sigma, while 4% paraformaldehyde (PFA) was provided by Beijing Solarbio Science & Technology Co., Ltd., and ethylenediaminetetraacetic acid (EDTA) decalcification solution was purchased from Wuhan Servicebio Biotechnology Co., Ltd.

**Synthesis of PST bone waxes:** resorbable bone wax samples with varying component ratios were prepared according to the formulation specified in Table S1. Firstly, pregelatinized QS was thoroughly mixed with β-TCP through vigorous agitation. Subsequently, the homogeneous powder mixture was incorporated into a preheated (70℃) polymer dispersion matrix composed of PEG-PPG and P188, followed by mechanical stirring to ensure uniform dispersion. The resulting slurry was then injected into molds for cooling and solidification. Finally, all bone wax samples were sterilized using gamma irradiation (25-40 kGy) prior to subsequent experimental procedures.

**Morphological characterization:** Scanning electron microscopy (SEM, Zeiss Ultra-55, Germany) was employed to observe the spherical microstructure of QS and the surface morphology of PST bone waxes.

**Particle size and zeta potential:** The introduction of quaternary ammonium groups in QS was confirmed by hydrogen nuclear magnetic resonance spectroscopy (^1^H NMR). Subsequently, the particle size distribution and surface zeta potential of QS, β-TCP, QS/β-TCP and solution after PST bone waxes dissolved were measured using a laser particle size analyzer (Zetasizer Nano ZS, Malvern Instruments, UK) to comprehensively evaluate its physicochemical characteristics.

**Texture analysis:** Performed using a universal testing machine (ELF3200, Bose, USA) under controlled conditions of 25℃ and 50% relative humidity. Spherical bone wax samples with a mass of 0.3 g and diameter of 8 mm were prepared. To simulate the resistance of materials under finger pressure during kneading, samples were placed between two compression plates and compressed to 3 mm deformation at a crosshead speed of 1 mm·min⁻¹. The compression stiffness was calculated based on the initial slope of the load/deformation curve to characterize the elastic properties of the material, which corresponds to the initial response of the sample prior to kneading in handling property tests. Subsequently, the testing mode was switched to tension mode, and the plates were separated at the same speed until sample detachment. The maximum adhesion load, maximum adhesion deformation, and adhesion energy were measured to comprehensively evaluate the adhesive performance between the sample and the plates^[1,2]^. PST bone waxes were manually softened by finger kneading to achieve plasticity, then filled into medullary cavity molds.

***In vitro* simulated sealing test:** To preliminarily evaluate the hemostatic efficacy of bone wax in sealing bone drilling defects *in vivo*, a liquid sealing test was designed as an *in vitro* assessment method. Specifically, the prepared bone wax sample was used to seal one end of an acrylic tube with a diameter of 3 mm. Subsequently, water was injected into the other end of the tube until the liquid level reached a height of 1.91 m (corresponding to a pressure of 18.68 kPa, equivalent to 140 mmHg, simulating intravascular pressure^[3]^), and the time until leakage occurred at the sealed area was continuously monitored.

***In vitro* resorption and mineralization:** PST bone waxes samples were immersed in PBS solution (37℃, 80 rpm), with complete dissolution time recorded in real-time and morphological changes documented via digital photography. Fresh PBS solution was replaced every 24 hours, and residual samples were collected to calculate the residual mass (RM) using Equation (1):

RM = (W1/W0)×100% (1)

where W0 represents the initial mass of bone wax and W1 denotes the remaining mass after dissolution. Simultaneously, inductively coupled plasma mass spectrometry (ICP-MS, Thermo iCAP 7000 series, USA) was employed to quantitatively analyze Ca²⁺/PO₄³⁻ release in supernatants over 21 days. Fourier transform infrared (FT-IR) spectroscopy was applied to evaluate resorption of the polymeric dispersion matrix by detecting supernatant components. The pH variation of PBS solution after bone wax deposition was monitored using a pH meter (PHS-25, Shanghai INESA Scientific Instrument Co., Ltd.). Furthermore, SEM-EDS analysis was performed on residual samples collected at 1, 7, 14, and 21 days to characterize micro-morphology and elemental distribution. X-ray diffraction (XRD, Thermo Scientific K-α+, UK) was utilized to assess mineralization patterns, with phase composition identified by comparing diffraction peaks to the hydroxyapatite (HA, PDF 09-0432) standard card. The crystallite size (D) was calculated via the Scherrer equation^[4]^ (2):

D = (κ×λ)/(FWHM(2θ)×cosθ) (2)

where κ is the shape factor (0.9), λ represents the Cu-Kα radiation wavelength (0.154 nm), and FWHM(2θ) corresponds to the full width at half maximum at 2θ=26°.

**Cell compatibility:** Bone marrow-derived mesenchymal stem cells (BMSCs) were isolated from Sprague-Dawley (SD) rats. Sterilized bone wax samples were immersed in DMEM medium to prepare extracts, which were then mixed with complete culture medium containing 10% FBS and 1% penicillin-streptomycin. BMSCs were cultured in this medium at 37℃ in a 5% CO₂ incubator, with fresh bone wax extracts replaced every 2-3 days to maintain culture stability. Cell viability was assessed on days 1, 3, 5, and 7 using the Cell Counting Kit-8 (CCK-8 assay, Beyotime Biotechnology, Shanghai, China), with absorbance measured at 450 nm using a microplate reader (Synergy H1, BioTek, USA) to quantify proliferation. Simultaneously, live/dead cell staining (Keygen Biotech, Jiangsu, China) was performed on days 1, 3, and 5, and fluorescence microscopy (MF50, MSHOT, Guangzhou, China) was used to capture images for evaluating cell survival status. Additionally, a scratch wound assay was conducted to monitor cell migration area over 48 hours, systematically assessing BMSCs migratory capacity in the bone wax extract microenvironment.

**Osteogenic differentiation:** BMSCs were cultured in bone wax extracts containing osteogenic induction components (50 μM ascorbic acid, 10 mM β-glycerophosphate, 100 nM dexamethasone), with the medium refreshed every 2-3 days to maintain the differentiation microenvironment. Alkaline phosphatase (ALP) activity was evaluated on days 7 and 14 using an ALP assay kit (Beyotime Biotechnology, Shanghai, China) and BCIP/NBT chromogenic substrate kit (Beyotime Biotechnology, Shanghai, China). After 21 days of culture, extracellular matrix mineralization was assessed via Alizarin Red S (ARS) staining following the manufacturer's protocol, followed by semi-quantitative analysis of calcium nodules through dissolution in 10% CTAC and OD measurement at 562 nm^[5]^.

Total RNA was extracted after 14 days of culture for RT-PCR analysis of osteogenesis-related genes, including osteocalcin (OCN), osteopontin (OPN), runt-related transcription factor 2 (RUNX2), ALP, and collagen type I (Col I). Gene expression levels were normalized to the control group (cells cultured in standard plates) using primer sequences listed in Table S2.

For protein localization, BMSCs seeded on confocal dishes were cultured in bone wax extracts for 14 days, followed by sequential processing: fixation with 4% paraformaldehyde (RT, 15 min), permeabilization with 0.1% Triton X-100 (Beyotime Biotechnology, Shanghai, China) for 10 min, blocking with 1% BSA (Biosharp, China) for 30 min, overnight incubation with OPN primary antibody (1:200 dilution, Proteintech), secondary antibody (1:500 dilution, Proteintech) protected from light and incubation for 1 h, and DAPI nuclear counterstaining (Beyotime Biotechnology, Shanghai, China, 5 min). OPN protein expression was visualized using a laser confocal microscope (Zeiss LSM 900).

**Hemolysis assay:** Whole blood was collected from male SD rats (200-250 g, Experimental Animal Center, Southern Medical University) using 3.8% sodium citrate anticoagulant tubes. The blood was centrifuged at 3000 rpm for 15 minutes at 4℃ to separate red blood cells (RBCs) from platelet-rich plasma (PRP). The RBC pellet was washed three times with PBS and resuspended in saline to prepare a 2% (v/v) erythrocyte suspension^[6]^.

The RBCs suspension was co-incubated with different bone wax samples, deionized water containing 1% Triton X-100 (TX-100, positive control), and PBS (negative control) at 37℃ for 3 hours. After centrifugation at 3000 rpm, the supernatant was collected for absorbance measurement at 545 nm (Abs)^[6]^. The hemolysis rate was calculated using Equation (3):

Hemolysis rate (%) = (Abst - Absn)/(Absp - Absn) × 100% (3)

where Abst, Absn, and Absp represent the absorbance values of the test group, negative control, and positive control, respectively.

**RBCs aggregation assay:** Five milligrams of bone wax samples (experimental group) or gauze (control group) were evenly placed in round-bottomed tubes. Premixed anticoagulated whole blood diluted 50% (270 μL) was rapidly combined with 0.1 M CaCl₂ solution (30 μL) and immediately transferred into the tubes. The tubes were incubated in a shaking incubator at 37℃ and 80 rpm for different time points (1, 5, 10, and 20 minutes). At each time point, the reaction was terminated by adding 5 mL of deionized water to lyse non-aggregated RBCs. The mixture was then transferred to centrifuge tubes and centrifuged at 1,000 rpm for 5 minutes. The supernatant was collected for hemoglobin absorbance measurement at 540 nm using a microplate reader.

Simultaneously, the precipitate was collected and unattached red blood cells were washed three times with PBS buffer. RBCs adhering to the bone wax surface were fixed with 2% glutaraldehyde solution for 12 hours, followed by gradient dehydration in 50%, 70%, and 90% ethanol (10 minutes each). The samples were then freeze-dried and observed under a SEM to compare the morphological differences of RBCs adhesion between bone wax and gauze surfaces.

**Platelets adhesion/activation assay:** PRP was gradient-diluted with PBS and quantified using a cell counter. 5 mg of bone wax samples or gauze (control group) were incubated with 100 μL of diluted PRP in plastic centrifuge tubes at 37℃ for 30 minutes. After incubation, the supernatant was discarded, and unattached platelets were removed by PBS washing. Samples were transferred to new centrifuge tubes and lysed with 250 μL of 1% TX-100 solution at 37℃ for 1 hour to release adherent platelets. The number of adherent platelets was quantified by measuring lactate dehydrogenase (LDH) activity in the lysate using an LDH assay kit (Shanghai Dongren Chemical Technology Co., Ltd., China). Simultaneously, the precipitates after PRP incubation were processed for SEM analysis following the platelets adhesion observation protocol to compare the micro-morphology of platelets adhesion and activation on bone wax and gauze surfaces. Furthermore, intracellular calcium ion concentration was measured to assess platelet activation: PRP (500 μL) was centrifuged at 3800 rpm for 15 minutes to remove supernatant, followed by resuspension in Fluo-4 AM calcium probe (Beyotime Biotechnology, Shanghai, China) and incubation at 37℃ for 15 minutes in the dark. Bone wax samples (10 mg) were then added, and the mixture was further incubated for 15 minutes under dark conditions. Fluorescence intensity was measured using a microplate reader (excitation wavelength: 494 nm; emission wavelength: 516 nm) after transferring 100 μL of supernatant to a 96-well plate, with results reflecting platelet activation levels.

***In vitro* blood clotting index (BCI):** 10 μL of whole blood were pipetted into plastic centrifuge tubes containing 5 mg of different bone wax samples or gauze (control group), followed by immediate addition of 1 μL of 0.2 M CaCl₂ (Beyotime Biotechnology, Shanghai, China) aqueous solution (triplicate samples per group). The mixture was incubated at 37℃ for 5 minutes to initiate coagulation. Subsequently, 2 mL of deionized water was added to lyse non-coagulated RBCs, followed by another 5-minute incubation at 37℃ to ensure complete hemolysis. The reaction system was then centrifuged at 500 rpm for 1 minute to separate phases, and the supernatant was collected for detection. Hemoglobin (HGB) content in each group was determined by measuring absorbance at 520 nm (Abssample). A negative control (Absblank) was established using 10 μL of citrate-anticoagulated whole blood dissolved in 2 mL deionized water. The same procedure was repeated for PPP treated without CaCl₂ to determine baseline BCI values^[6]^. Finally, the BCI was calculated using Equation (4):

BCI = (Abssample/Absblank) × 100% (4)

**APTT/PT assays:** Fresh citrate-anticoagulated whole blood was collected from SD rats and centrifuged at 3000 rpm for 15 minutes at 4℃ to obtain platelet-poor plasma (PPP). The coagulation times of PPP in contact with bone wax materials were measured following the standard protocols of the activated partial thromboplastin time (APTT) assay kit and prothrombin time (PT) assay kit (Shanghai Yuanye Bio-Technology Co., Ltd.), systematically evaluating the material's impact on intrinsic and extrinsic coagulation pathways^[7]^.

**Ethics and animal model establishment:** This study systematically evaluated the *in vivo* hemostatic efficacy and bone regeneration performance of different bone waxes using tibial bone defect models in New Zealand white rabbits and beagle dogs. All animal experiments were conducted at the Guangdong Laboratory Animals Monitoring Institute and strictly adhered to protocols approved by the institution's Institutional Animal Care and Use Committee (IACUC) under approval numbers IAC24W130 (15) and No.2024107.

**Rabbit cancellous bone defect model:** 36 male New Zealand white rabbits (2-2.5 kg) were randomly assigned to groups and anesthetized via intravenous injection of 3% pentobarbital sodium solution (40 mg/kg, Merck KGaA, Germany). After shaving and disinfecting the right hind limb, a skin-muscle incision was made, and a cylindrical bone hole (diameter 4.2 mm, depth 3 mm) was created in the medial tibia using a circular chisel to establish a chronic/penetrating cancellous bone hemorrhage model. Bone wax was applied to the defect site for hemostasis, with bleeding volume recorded within 3 minutes and digital photographs captured to document wound status. Wounds were then sutured. To evaluate hemorrhage dynamics during pre-healing, venous blood samples were collected on postoperative days 1, 4, and 7 for complete blood count analysis.

At experimental endpoints (weeks 4 and 8), rabbits were euthanized, and the right tibia was harvested and fixed in 4% paraformaldehyde. Micro-computed tomography (micro-CT, Aloka Latheta LCT200, Hitachi, Japan) was performed on the repair region, with a circular region of interest (ROI, diameter 5 mm) above the defect analyzed for bone mineral density (BMD), bone volume fraction (BV/TV), trabecular number (Tb.N), and trabecular thickness (Tb.Th). After EDTA decalcification, specimens underwent histological processing including hematoxylin-eosin (H&E) staining, Masson's trichrome staining, and immunohistochemical analysis for OCN, OPN, Runx2, interleukin-1β (IL-1β), and tumor necrosis factor-α (TNF-α).

**Beagles cancellous bone defect model:** 12 male beagle dogs (10-15 kg, purchased from Zhenjiang Wanwei Experimental Animal Breeding Co., Ltd.) were housed under specific pathogen-free (SPF) conditions after passing quality inspections. Animals were randomly divided into experimental and control groups, then anesthetized via intravenous injection of 3% pentobarbital sodium solution (0.8 mL/kg). Following anesthesia, three cylindrical bone defects (diameter 4 mm × depth 6 mm) were created bilaterally in the tibiae using standardized procedures identical to those established for rabbit tibial defect models. Immediately after defect creation, bone wax was applied to the wounds for hemostasis, with intraoperative bleeding volume recorded within 3 minutes and digital photographs captured for documentation. Skin and muscle incisions were routinely sutured, and postoperative ambulation recovery was monitored during wound healing.

To evaluate early-stage hemorrhage dynamics, wound exudates (tissue fluid) were collected via syringe at defined intervals within the first postoperative week, with volumetric measurements recorded. At experimental endpoints (6 and 12 weeks post-surgery), dogs were euthanized, and bilateral tibiae were harvested and fixed in 4% paraformaldehyde. Micro-CT was employed for three-dimensional quantitative imaging analysis, followed by standard EDTA decalcification and paraffin embedding. Histological sections were prepared for H&E staining and Masson's trichrome staining to assess bone repair outcomes.

**Statistical analysis:** Each experiment was repeated at least three times (n≥3), and all data are presented as mean ± standard deviation (SD). Statistical analysis of the data was carried out by origin 2022 software, and the relevant data of *in vitro* and *in vivo* experiments were analyzed by one-way ANOVA (One-way ANOVA) analysis. When p<0.05 (*), p< 0.01 (**) and p<0.001 (***), statistical significance was defined.

**Table S1** Different compositions of resorbable bone wax.

| Materials  Group | QS | β-TCP | P188 | PEG-PPG |
| --- | --- | --- | --- | --- |
| PT1 | 0 | 3.5% | 37% | 59.5% |
| PST1 | 3.5% | 3.5% | 37% | 56% |
| PST2 | 3.5% | 7.5% | 37% | 52% |
| PST3 | 3.5% | 10.5% | 37% | 49% |

**Table S2.** Primers sequences used for RT-PCR^[5]^.

| **Gene** | **Forward sequence** | **Reverse sequence** |
| --- | --- | --- |
| OCN  OPN | ATGGAGCCCTCACACTCCT  GCTTCCAGCAGACATCACCA | CTTGACACAAAGGCTGCAC  TGGTGGTGGTGGTGGTGGTG |
| COL-1 | GACATGTTCAGCTTTGTGGACCTC | AGGGACCCTTAGGCCATTGTGTA |
| RUNX-2 | CATGGCCGGGAATGATGAG | TGTGAAGACCGTTATGGTCAAAGTGATG |
| ALP | CATCGCCTATCAGCTAATGCACA | AGGTCCAGGCCATCCAG |
| GADPH | GGCACAGTCAAGGCTGAGAATG | ATGGTGGTGAAGACGCCAGTA |

**2. Fig. Section**


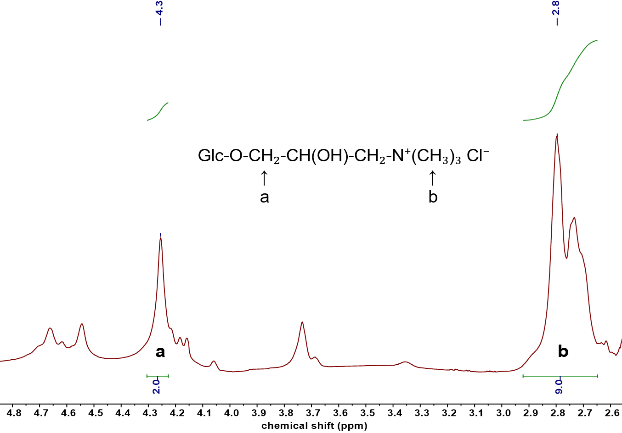


**Fig. S1.** ^1^H NMR spectrum of QS.

**Analyze：**The ^1^H NMR spectrum of QS revealed the structural features of the quaternized starch derivative. QS is constructed from α-D-glucopyranose units (Glc) linked by α-1,4-glycosidic bonds, with hydroxypropyl trimethylammonium groups introduced on the hydroxyl moieties of the glucose units^[8]^. The chemical shift around 4.3 ppm corresponds to the (a) Glc-O-CH_2_ protons associated with the glycosidic linkages in the starch backbone. Meanwhile, the signal near 2.8 ppm is attributed to the (b) N^+^(CH_3_)_3_ methyl groups, indicative of the quaternary ammonium moieties^[6]^. These findings confirm the successful modification of starch with quaternary ammonium groups.


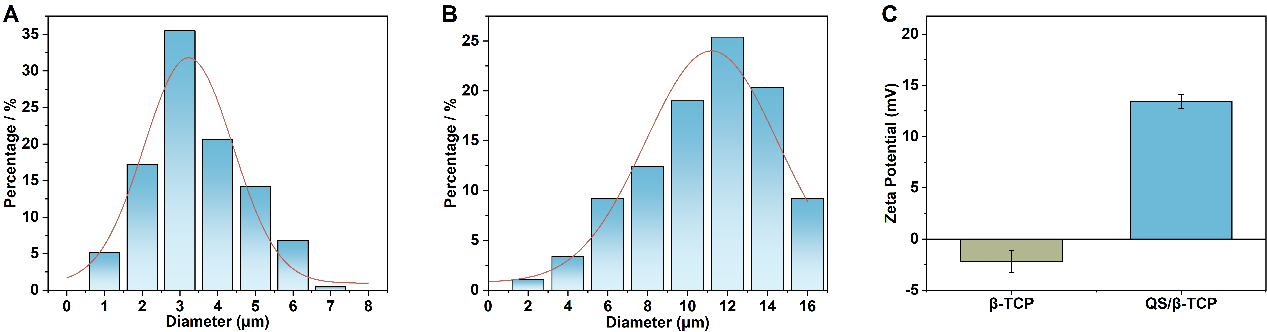


**Fig. S2.** (A) Size distribution of β-TCP and (B) QS/β-TCP composite particles. (C) Zeta potential of β-TCP and QS/β-TCP composite particles.


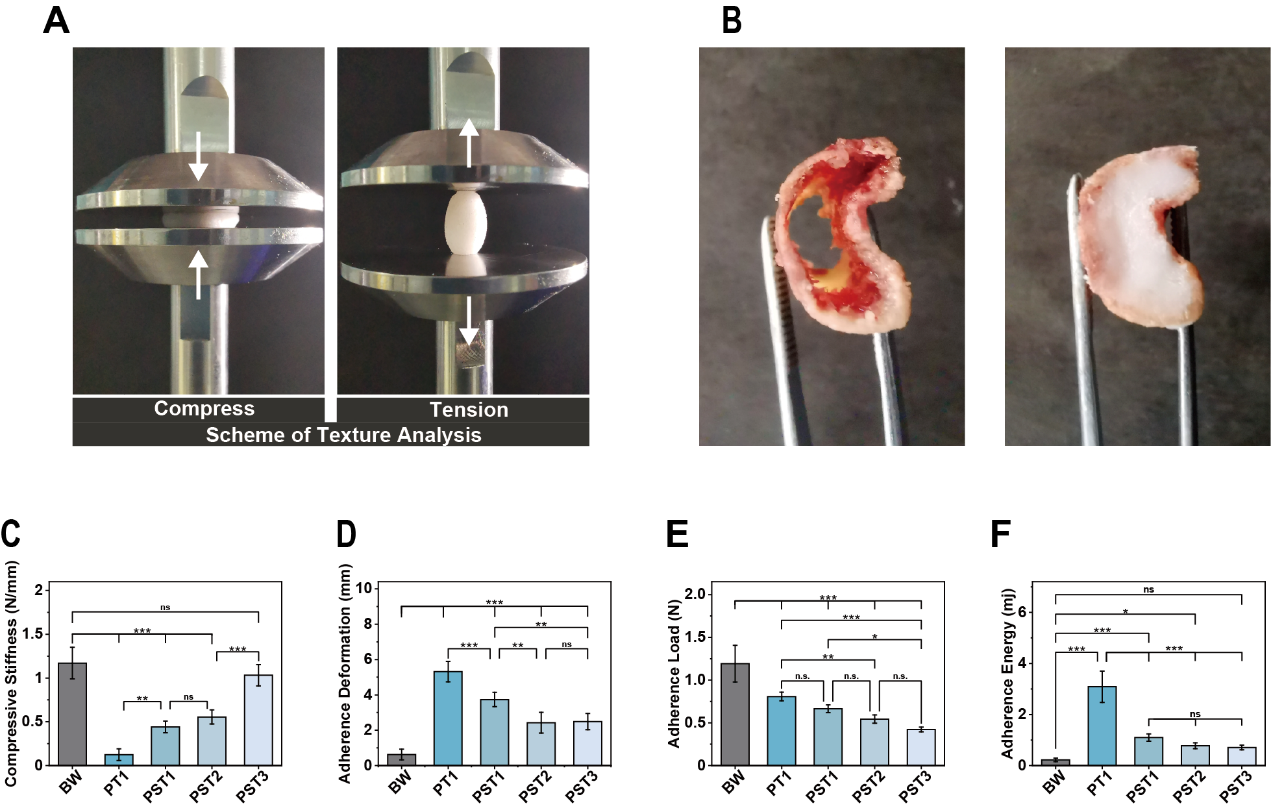


**Fig. S3.** **Texture analysis of bone wax.** (A) Texture analysis schematic. (B) Plasticizing properties of PST bone wax. (C) Compressive stiffness of bone wax from the texture analysis curve (BW: Johnson & Johnson Commercial Bone Wax). (D) Adherence deformation of bone wax from the texture analysis curve. (E) Adherence load of bone wax from the texture analysis curve. (F) Adherence energy of bone wax from the texture analysis curve. n ≥ 3 for **each** group. Error bars denote means ± SD; *P < 0.05, **P < 0.01, and ***P < 0.001.

**
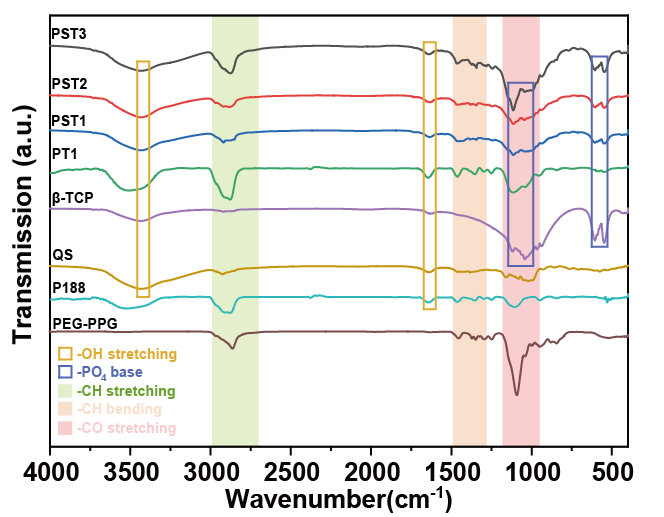
**

**Fig. S4.** FT-IR spectra of different bone wax materials.


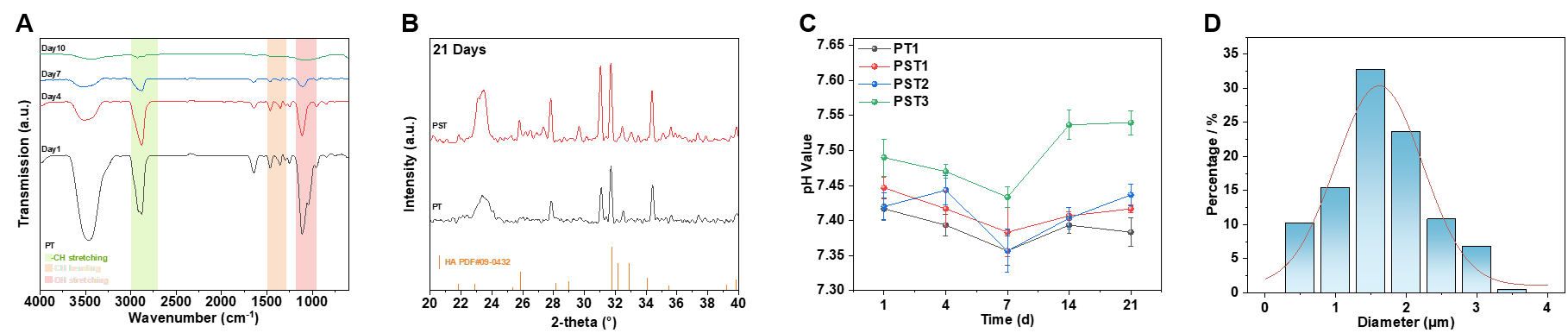


**Fig. S5.** Resorption **behavior of synthesized absorbable bone wax.** (A) FT-IR spectra of PT bone wax during 10 days of resorption. (B) XRD of PT and PST bone wax at 21 days of resorption, **characteristic** peaks of hydroxyapatite (PDF Ref. 09-0432). (C) The pH changes in bone wax deposition in PBS. (D) Particle size distribution of free particles in the solution after PST completely dissolved. n ≥ 3 for each group. Error bars denote means ± SD.


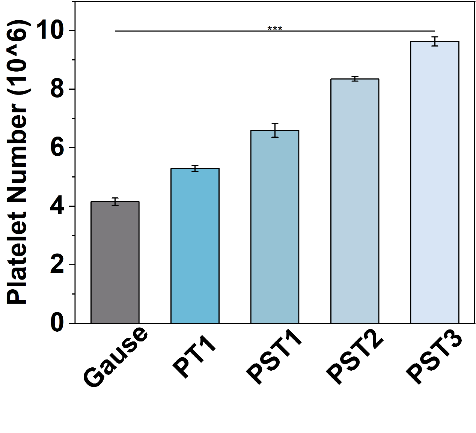


**Fig. S6.** Adherent platelet number treated with PST bone waxes. n ≥ 3 for each group. Error bars denote means ± SD; *P < 0.05, **P < 0.01, and ***P < 0.001.


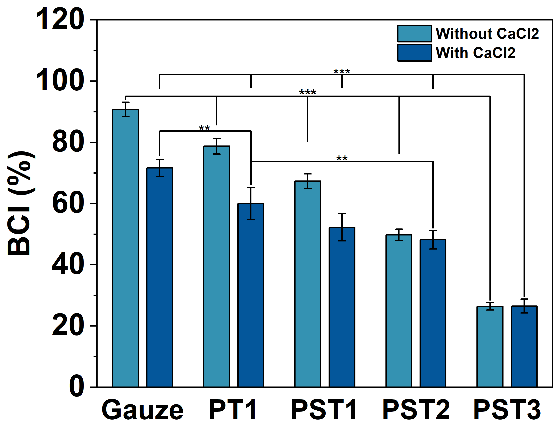


**Fig. S7.** *In vitro* blood clotting index (BCI). n ≥ 3 for each group. Error bars denote means ± SD; *P < 0.05, **P < 0.01, and ***P < 0.001.


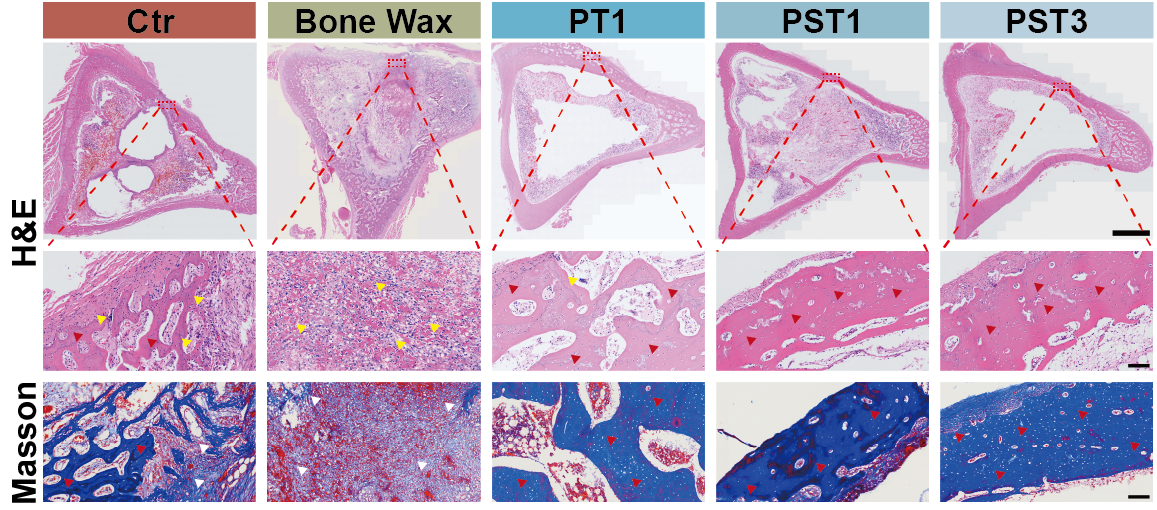


**Fig. S8.** H&E staining of bone sections after 8 weeks of treatment with different bone waxes (scale bar, 1000 μm) and magnified views of the defect area (scale bar, 100 μm; yellow triangles, inflammatory cells; red triangles, new bone tissue). Masson trichrome staining of bone sections after 8 weeks (scale bar, 100 μm; white triangles, myofibers).


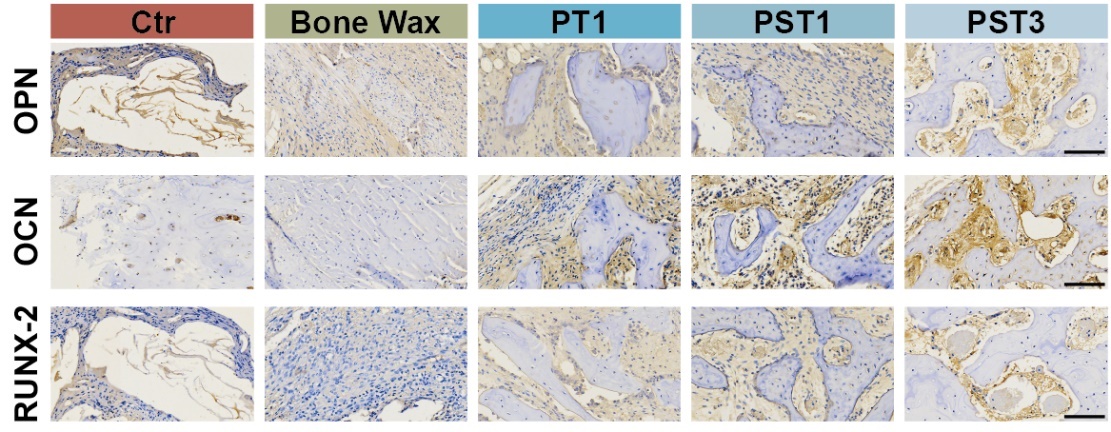


**Fig. S9.** Immunohistochemical staining of bone sections after 4 weeks of treatment.


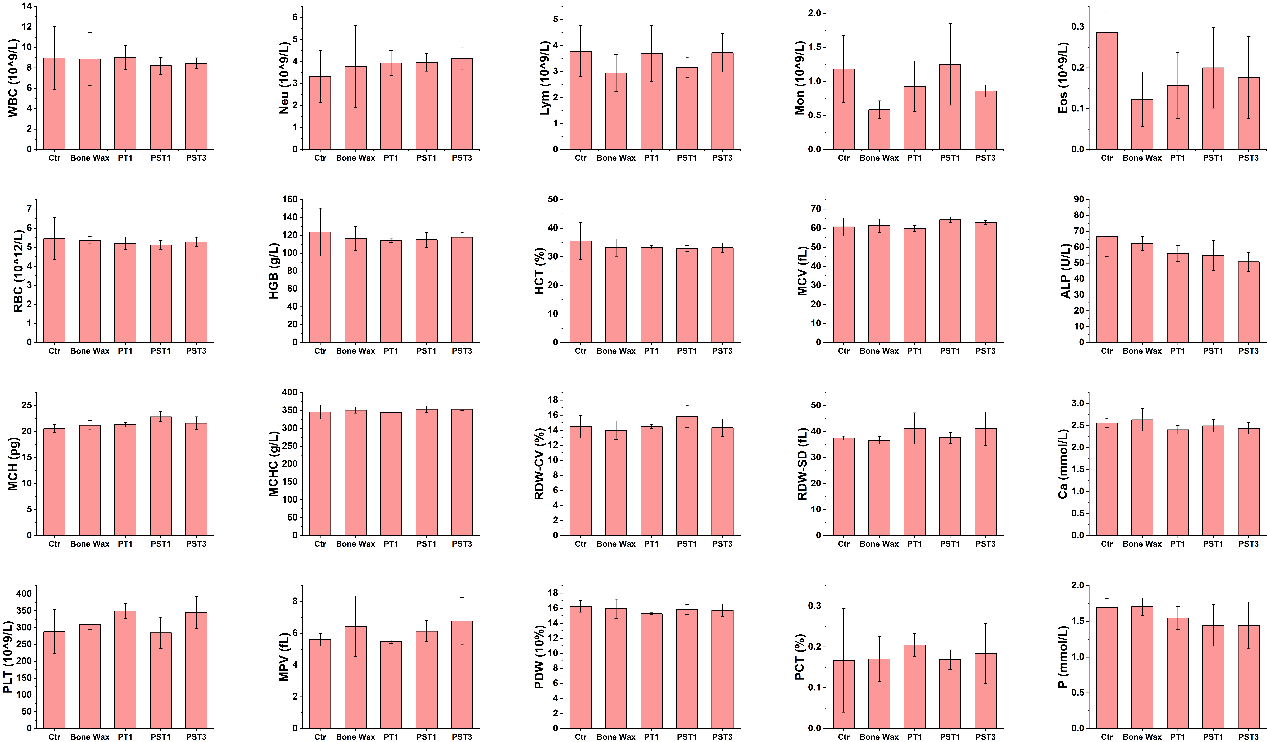


**Fig. S10.** **Hematological and biochemical indicators in rabbits after bone wax implantation for 8 weeks.** n ≥ 3 for each group. Error bars denote means ± SD; *P < 0.05, **P < 0.01, and ***P < 0.001.


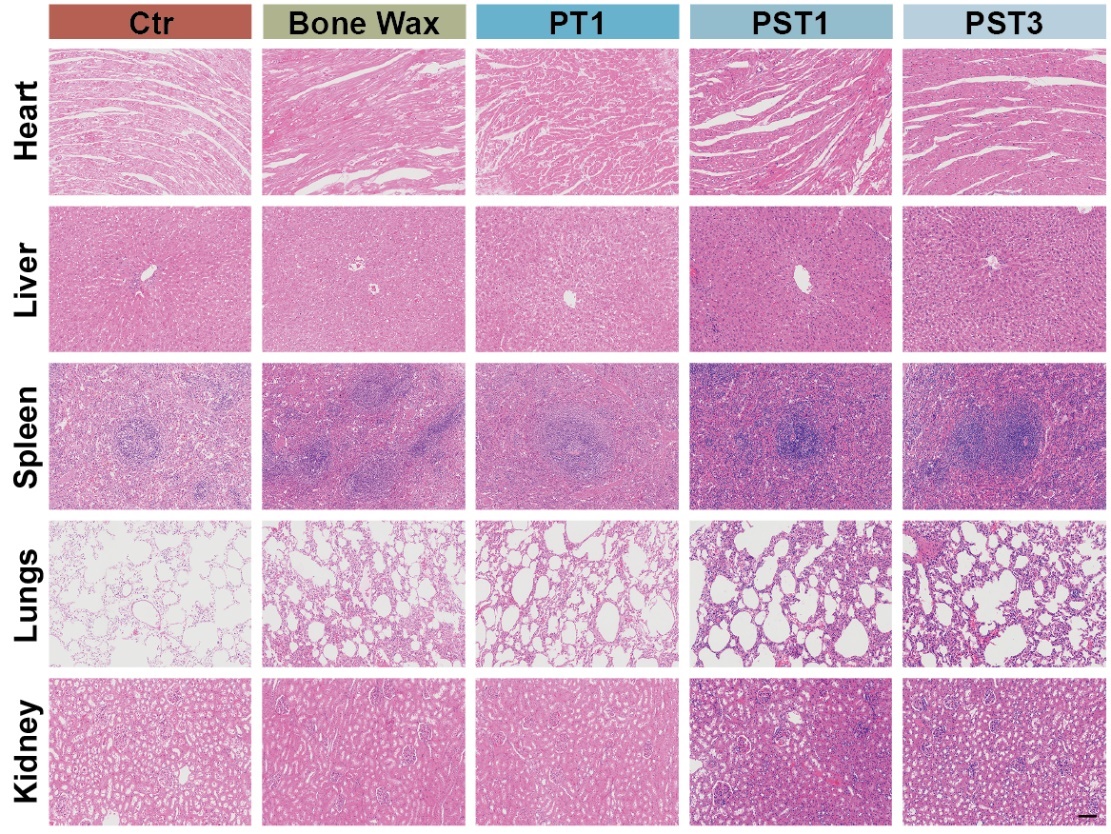


**Fig. S11.** H&E staining of major organs after bone wax implantation for 8 weeks.


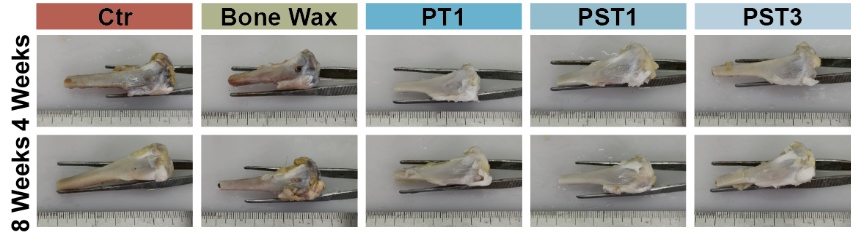


**Fig. S12.** **Tibial defect sites in rabbits after bone wax implantation for 4 and 8** weeks**.**

**Fig. S13.** Blood loss within 3 minutes of bone wax application at the defect site. n ≥ 3 for each group. Error bars denote means ± SD; *P < 0.05, **P < 0.01, and ***P < 0.001.


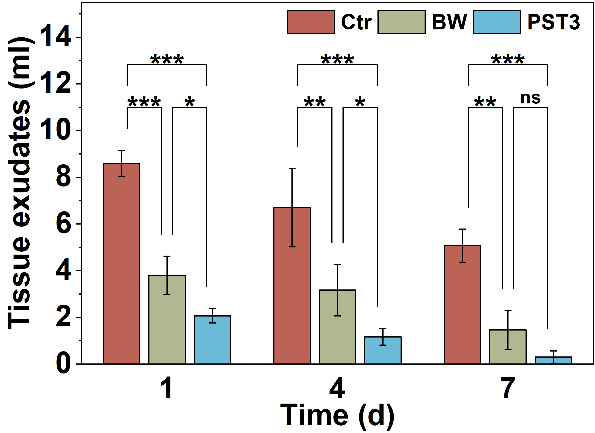


**Fig. S14.** Wound tissue exudation in Beagles. n ≥ 3 for each group. Error bars denote means ± SD; *P < 0.05, **P < 0.01, and ***P < 0.001.


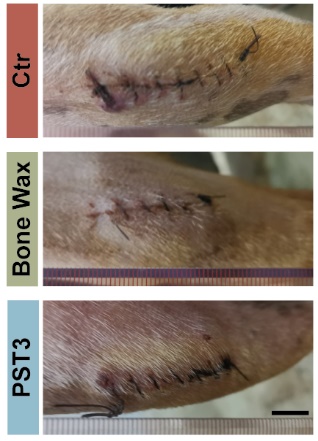


**Fig. S15.** Photograph of Beagle wound tissue showing erythema, swelling, and exudate at 1 week post-operation (Scale bar, 1 cm).


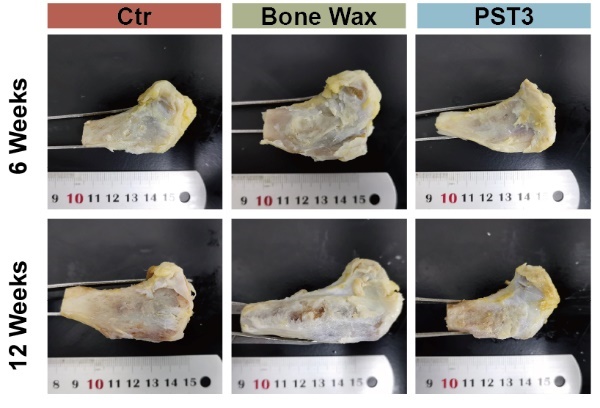


**Fig. S16.** **Tibial defect sites in** beagles **after bone wax implantation for 6 and 12 weeks.**

**References**

[1] Yao L, Lio J, Wang T, et al. Synthesis and characterization of acetylated and stearylyzed soy wax[J]. Journal of the American Oil Chemists’ Society, 2013, 90(7): 1063–1071.

[2] Suwanprateeb J, Suvannapruk W, Thammarakcharoen F, et al. Preparation and characterization of peg–ppg–peg copolymer/pregelatinized starch blends for use as resorbable bone hemostatic wax[J]. Journal of Materials Science: Materials in Medicine, 2013, 24(12): 2881–2888.

[3] Tan X, Gerhard E, Wang Y, et al. Development of biodegradable osteopromotive citrate-based bone putty[J]. Small, 2022, 18(36): 2203003.

[4] Brückner T, Schamel M, Kübler A C, et al. Novel bone wax based on poly(ethylene glycol)–calcium phosphate cement mixtures[J]. Acta Biomaterialia, 2016, 33: 252–263.

[5] Zhang Y, Wang H, Huangfu H, et al. 3D printing of bone scaffolds for treating infected mandible bone defects through adjustable dual-release of chlorhexidine and osteogenic peptide[J]. Materials & Design, 2022, 224: 111288.

[6] Liu J, Hu Y, Li L, et al. Biomass‐derived multilayer‐structured microparticles for accelerated hemostasis and bone repair[J]. Advanced Science, 2020, 7(22): 2002243.

[7] Tong L, Zhang D, Huang Z, et al. Calcium ion-coupled polyphosphates with different degrees of polymerization for bleeding control[J]. ACS Applied Materials & Interfaces, 2024, 16(33): 43244-43256.

[8] Ribeiro De Carvalho G, Kudaka A M, Fares Sampar J, et al. Quaternization of cassava starch and determination of antimicrobial activity against bacteria and coronavirus[J]. Carbohydrate Research, 2024, 538: 109098.
